# Centrosomal Localization of the Psoriasis Candidate Gene Product, *CCHCR1*, Supports a Role in Cytoskeletal Organization

**DOI:** 10.1371/journal.pone.0049920

**Published:** 2012-11-26

**Authors:** Mari H. Tervaniemi, H. Annika Siitonen, Cilla Söderhäll, Gurinder Minhas, Jyrki Vuola, Inkeri Tiala, Raija Sormunen, Lena Samuelsson, Sari Suomela, Juha Kere, Outi Elomaa

**Affiliations:** 1 Haartman Institute, Department of Medical Genetics, University of Helsinki, Helsinki, Finland; 2 Research Program's Unit, Molecular Medicine, University of Helsinki, Helsinki, Finland; 3 Folkhälsan Institute of Genetics, Helsinki, Finland; 4 Department of Biosciences and Nutrition, Karolinska Institutet, Stockholm, Sweden; 5 Helsinki Burn Centre, Department of Plastic Surgery, Helsinki University Central Hospital, Helsinki, Finland; 6 Biocenter Oulu, Department of Pathology, University of Oulu, Oulu, Finland; 7 Department of Clinical Genetics, Sahlgrenska University Hospital, Gothenburg, Sweden; 8 Department of Dermatology, University of Helsinki, and Helsinki University Central Hospital, Helsinki, Finland; 9 Science for Life Laboratory, Karolinska Institutet, Stockholm, Sweden; Tor Vergata University of Rome, Italy

## Abstract

*CCHCR1* (Coiled-Coil α-Helical Rod protein 1), within the major psoriasis susceptibility locus *PSORS1*, is a plausible candidate gene with the psoriasis associated risk allele CCHCR1*WWCC. Although its expression pattern in psoriatic skin differs from healthy skin and its overexpression influences cell proliferation in transgenic mice, its role as a psoriasis effector gene has remained unsettled. The 5′-region of the gene contains a SNP (rs3130453) that controls a 5′-extended open reading frame and thus the translation of alternative isoforms. We have now compared the function of two CCHCR1 isoforms: the novel longer isoform 1 and the previously studied isoform 3. In samples of Finnish and Swedish families, the allele generating only isoform 3 shows association with psoriasis (*P*<10^−7^). Both isoforms localize at the centrosome, a cell organelle playing a role in cell division. In stably transfected cells the isoform 3 affects cell proliferation and with the CCHCR1*WWCC allele, also apoptosis. Furthermore, cells overexpressing CCHCR1 show isoform- and haplotype-specific influences in the cell size and shape and alterations in the organization and expression of the cytoskeletal proteins actin, vimentin, and cytokeratins. The isoform 1 with the non-risk allele induces the expression of keratin 17, a hallmark for psoriasis; the silencing of CCHCR1 reduces its expression in HEK293 cells. CCHCR1 also regulates EGF-induced STAT3 activation in an isoform-specific manner: the tyrosine phosphorylation of STAT3 is disturbed in isoform 3-transfected cells. The centrosomal localization of CCHCR1 provides a connection to the abnormal cell proliferation and offers a link to possible cellular pathways altered in psoriasis.

## Introduction

Psoriasis is a chronic skin disease that affects 2–3% of people with European descent and is less common in other populations such as in Asia and Africa [1]. The most important chromosomal region for psoriasis predisposition is *PSORS1* in the Human Leukocyte Antigen region (HLA, 6p21.3) where also *CCHCR1* (Coiled-Coil α-Helical Rod protein 1), by position a plausible psoriasis candidate gene, is located [2], [3]. Even though *CCHCR1* resides in the chromosomal region showing the strongest associations in genome-wide association studies [4], its role and function in the pathogenesis of psoriasis is still unclear. The *CCHCR1* gene is highly polymorphic and has the allele CCHCR1*WWCC associated with psoriasis in several populations [2], [3], [5]. “WWCC” stands for the amino acids in the psoriasis risk haplotype, whereas in the non-risk haplotype the corresponding amino acids are RRGS. The CCHCR1 protein does not belong to any known protein family but is predicted to be a rod-like protein, with an alpha-helical coiled coil structure. The expression of CCHCR1 is different in psoriatic lesions when compared with healthy skin or other hyperproliferative skin disorders [6]. We and others have demonstrated that CCHCR1 regulates the synthesis of steroids from cholesterol in mitochondria by interacting with the steroidogenic activator protein StAR [7], [8]. Interestingly, a recent gene expression analysis revealed evidence for decreased lipid biosynthesis in uninvolved psoriatic skin, supporting the role of altered lipid metabolism in the pathogenesis of psoriasis [9]. Our recent findings showed that overexpression of CCHCR1 affects keratinocyte proliferation in transgenic mice. The most evident effect was observable after wounding and treatment with 12-O-tetradecanoyl-13-acetate (TPA); the number of proliferating keratinocytes was decreased and wound healing delayed in mice with the CCHCR1*WWCC risk allele [10]. Furthermore, the expression of several genes relevant in psoriasis pathogenesis were altered, these including cytokeratins 6, 16, and, 17, and genes of the epidermal differentiation complex region on the *PSORS4* locus (1q21), such as S100 calcium binding protein A1 (S100A) and small proline-rich protein (SPRR) [11].

As psoriasis and cancer share some characteristics, such as accelerated cell proliferation, angiogenesis, and inflammation, we have previously studied the expression of CCHCR1 in the non-melanoma skin cancers squamous cell carcinoma (SCC) and basal cell carcinoma (BCC) [12]. In these tumors, unlike in psoriasis, CCHCR1 is expressed especially in proliferating cells (Ki67 positive). Furthermore, *CCHCR1* mRNA expression is upregulated in SCC cultures when compared to normal keratinocytes. Recently, a similar increase in CCHCR1 expression was observed in neoplastic cervical H-SIL samples (high-grade squamous intraepithelial lesions) [13]. Interestingly, the strongest CCHCR1 expression in SCCs and BCCs is observed in areas positive for epidermal growth factor receptor (EGFR). This is in agreement with the finding that EGF induces CCHCR1 expression in keratinocytes [8]. EGFR and its related receptors are well known markers in several solid tumors and their expression and signaling are implicated in psoriasis pathogenesis as well [14], [15], [16]. The persistent stimulation of EGFR was suggested to result in the constitutive activation of signal transducer and activator of transcription signal protein 3 (STAT3), having pathogenic effects in skin via alteration of biological processes, such as proliferation, differentiation, and apoptosis of keratinocytes [17], [18], [19], [20].

The centrosome determines the organization of the spindle poles during mitosis, therefore having a crucial function in cell division [21], [22]. It also plays a role in the organization of the microtubules and through its influence on the cytoskeleton it regulates cell shape, motility, and polarity. The centrosome consists of a pair of centrioles that are surrounded by a dense fibrillar network of proteins, called pericentriolar material (PCM). It comprises hundreds of proteins with many different functions, with γ-tubulin as a constitutive component. Defects in genes encoding centrosomal proteins, such as mitotic checkpoint genes, can cause abnormalities that are identifiable in most human cancer cells. For instance Aurora kinase A, a protein needed for the timely entry into mitosis, maturation of centrosomes, and assembly of bipolar spindles, is implicated in the development of epithelial cancers, such as SCC [23]. Also β-catenin, a molecule involved in Wnt pathway and cell-cell adhesion, was recently detected at the centrosome, where its phosphorylation regulates centrosome splitting and microtubule re-growth [24], [25], [26]. Alterations in β-catenin expression and Wnt signaling are observed in lesional psoriatic skin and also in cancers [27], [28].

We have cloned a novel isoform 1 of CCHCR1 where the N-terminal domain is 89 amino acids longer than in the previously studied isoform 3. The formation of the isoforms is dependent on a SNP (rs3130453) that with alleles G and A results in either tryptophan (allele *Iso1*) or a stop codon (allele *Iso3*), respectively. Here we studied if either of the alleles *Iso1* or *Iso3* associates with psoriasis in family samples and whether the extended N-terminus of isoform 1 affects the localization and function of CCHCR1. By generating stable cell lines expressing either of the isoforms 1 and 3 with the non-risk (*RRGS*) or the risk haplotype (*WWCC*), we show that CCHCR1 has isoform- and allele specific effects in the cell. We hypothesize that an aberrant function of CCHCR1 might lead to abnormal keratinocyte growth, which is a key feature of the psoriatic epidermis.

## Results

### Cloning and expression of a novel CCHCR1 isoform 1

**Figure 1:**
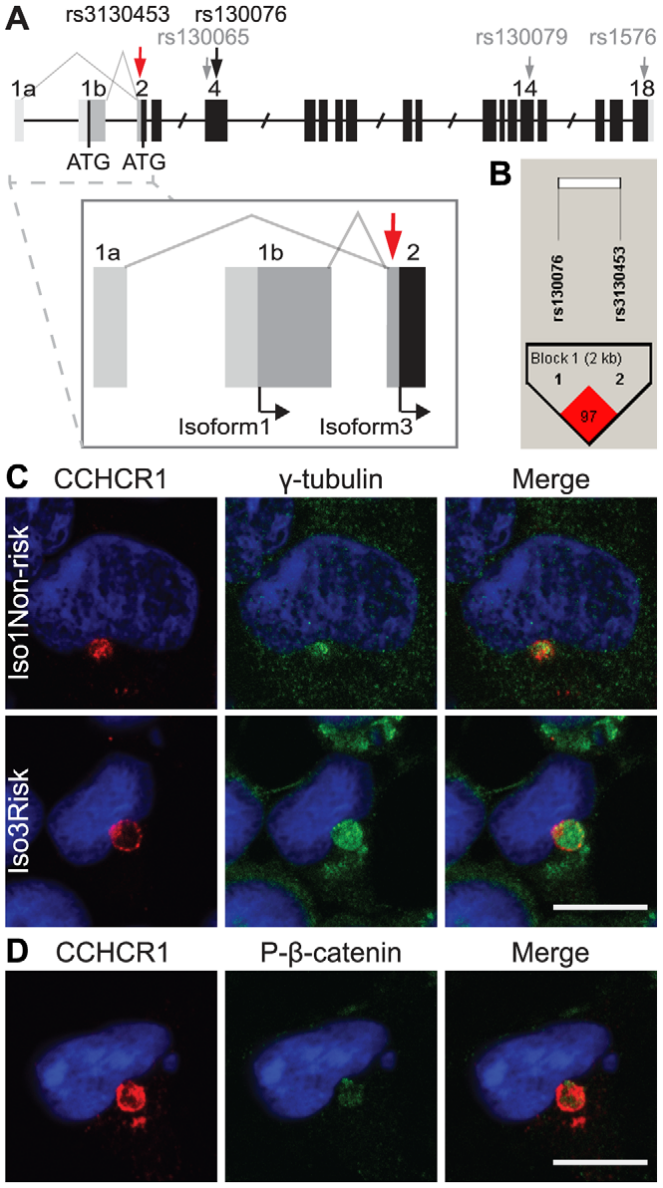
Alternative transcription of *CCHCR1* into transcripts 1 and 3 and localization to the centrosome. (**A**) The numbers represented above the schematic gene structure (not in scale) represent the exons. Transcripts 1 and 3, starting with either exon 1a (transcript 3) or 1b (transcript 1), result from alternative transcription start sites that are featured more clearly in the magnification of the 5′-region. The light grey color represents the untranslated region and the darker grey indicates the sequence for the additional 5′-region in the isoform 1, lacking from isoform 3. The red arrow in exon 2 indicates the SNP (rs3130453) with alleles G and A creating either tryptophan or stop codon, respectively. The former enables the usage of an earlier initiation codon (ATG) in the reading frame. The black and grey arrows show the positions of the *WWCC* haplotype SNPs in exons 4, 14, and 18. (**B**) The SNP regulating the 5′ open reading frame (rs3130453, *Iso1/Iso3) and one of the *WWCC SNPs (rs130076, second W) are in high linkage disequilibrium. (**C**) CCHCR1 localizes in the centrosomal area. Ds-Red-tagged CCHCR1 (red) shows co-localization with the centrosomal marker γ-tubulin (green) in stably transfected HEK293 cells. All CCHCR1 isoforms localize at the centrosome, here shown Iso1Non-risk and Iso3Risk. (**D**) CCHCR1 also co-localizes with the phosphorylated form of β-catenin at the centrosome. DAPI stained nuclei are shown in blue. Scale bars 10 µm.

A database survey suggested a putative longer isoform of CCHCR1. Based on NCBI's GenBank database, *CCHCR1* has alternative transcripts 1 and 3 (NM_001105564.1, NM_019052.3, respectively) resulting from two alternative transcription start sites (Fig. 1A) [7]. The previously studied CCHCR1 (HCR) cDNA [3], [11] corresponds to the transcript 3 starting with exon 1a and encoding for the shorter protein (isoform 3), whereas the transcript 1 starting with exon 1b is additionally able to encode for a longer protein (isoform 1) depending on a SNP (rs3130453) in exon 2. The SNP (G/A) creates either a codon for tryptophan (G) or a stop codon (A). The stop codon (allele *Iso3*) results in the shorter isoform whereas the codon for tryptophan (allele *Iso1*) enables the usage of an earlier translation start site in exon 1b, thus leading to a protein with 89 additional amino acids in its N-terminal domain. This N-terminal part shows no homology to any known proteins or protein motifs. To confirm the existence of both variants *in vivo*, we studied the mRNA expression of transcripts 1 and 3 of CCHCR1 in various human tissues and cell lines using RT-PCR. Both transcripts are detectable in all tissues and cells studied and do not show any major differences in their expression (Table S1). To study the function of CCHCR1 we cloned both variants into the pDsRed-tagged vector and generated HEK293 cell lines overexpressing either the isoform 1 or 3 with the risk (CCHCR1*WWCC) or the non-risk (CCHCR1*RRGS) haplotype (Fig. S1A and B). Lines are called: Iso1Risk, Iso1Non-risk, Iso3Risk, and Iso3Non-risk. We also established stable shRNA cell lines using HEK293 cells in which CCHCR1 expression is downregulated 50–60% (Fig. S1C). The HEK293 cells are homozygous for the *CCHCR1* non-risk and *Iso1* alleles and are therefore able to endogenously express also the isoform 1. A construct with scrambled sequence was used to prepare a control shRNA cell line.

### The SNP creating isoform 3 associates with psoriasis

To study the possible connection between the CCHCR1 isoforms and psoriasis, we genotyped the SNP rs3130453 (G/A) in 508 Finnish and Swedish psoriasis families (Table 1). The A allele resulting in the translation of only CCHCR1 isoform 3 (*Iso3* allele) shows preferential transmission from heterozygous parents to affected offspring (*P*<10^−7^). We also measured mRNA levels of transcripts 1 and 3 in skin samples from Finnish patients by using quantitative PCR (qPCR), but did not observe any differences in their expression (*P*>0.05) between non-lesional and lesional skin (data not shown). The mRNA amount of variant 1, however, is not directly comparable to the isoform 1 protein expression as transcript 1 is able to encode for the shorter protein isoform 3 as well. We also genotyped rs130076 (C/T), corresponding for the second R/W amino acid change in CCHCR1*WWCC, from the same family material. The risk allele *W showed association with psoriasis (*P*<10^−13^), as expected [2], [3]. Haplotype analysis with transmission disequilibrium test (TDT) showed undertransmission of *Iso1Non-risk (*P*<10^−7^) and the transmission of *Iso3Risk (*P*<10^−16^) to affected offspring, suggesting that the complete psoriasis risk allele is CCHCR1*Iso3WWCC (Table 2). The two markers are also in high linkage disequilibrium (LD) (Fig. 1B), as is the whole HLA-region within *PSORS1*.

**Table 1:** Transmission equilibrium of *Iso3 (rs3130453) and one of the Risk-allele *WWCC (rs130076) SNPs as single markers in psoriasis family samples of Finnish and Swedish origin.

SNP ID	HWpval	FamTrio	MAF	Alleles	Overtransm	T∶U	χ^2^	P-value
rs3130453	0.3895	531	0.461	A∶G	A	285∶168	30.219	3.86*10^−8^
rs130076	0.5943	497	0.273	C∶T	T	229∶91	59.512	1.22*10^−14^

Abbreviations: MAF, minor allele trequency; SNP, single-nucleotide polymorphism; HWpval, Hardy-Weinberg equilibrium P-value; T, transmitted; U, untransmitted. Significant P-values are shown in bold.

**Table 2:** Transmission equilibrium of *Iso3 (rs3130453) and one of the Risk-allele *WWCC (rs130076) SNPs as haplotypes in psoriasis family samples of Finnish and Swedish origin.

rs3130453	rs130076	Freq	T∶U	χ^2^	P-value	Effect	Description	
G	C	0.455	177.2∶292.9	28.463	9.55*10^−8^	Protective	Iso1	RRGS
A	T	0.274	266.6∶104.3	71.032	3.51*10^−17^	Risk	Iso3	WWCC
A	C	0.268	160.5∶203.1	4.995	0.03	Neutral	Iso3	RRGS

Abbreviations: SNP, single-nucleotide polymorphism; T, transmitted; U, untransmitted; Iso1, CCHCR1 isoform 1; Iso3, CCHCR1 isoform 3; RRGS, amino acids in non-risk haplotype; WWCC, risk haplotype. Significant P-values are shown in bold.

### CCHCR1 is localized at the centrosome

**Figure 2:**
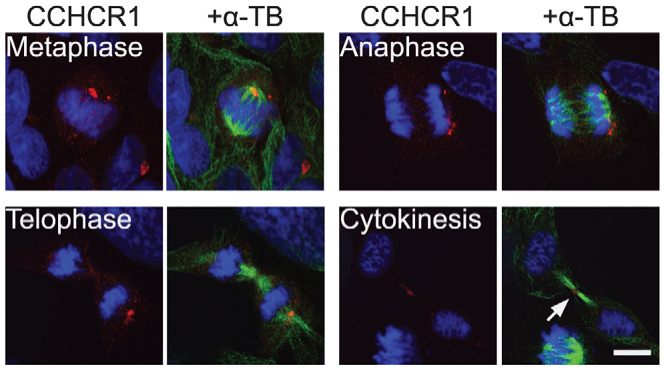
CCHCR1 protein is expressed throughout the cell cycle but the localization is dynamic. Ds-Red-tagged CCHCR1 (red) is stably expressed and the cells are stained with α-tubulin (α-TB, green) antibodies for the detection of different points of the cell cycle (metaphase, anaphase, telophase and cytokinesis). Near the end of cytokinesis CCHCR1 is also detectable at the midbody region (arrow) connecting the dividing cells. DAPI stained nuclei are shown in blue. Scale bar 10 µm.

In stable HEK293 cell lines, all of the Iso1Risk, Iso1Non-risk, Iso3Risk, and Iso3Non-risk DsRed-tagged CCHCR1 constructs localize at the centrosome, showing overlapping or adjacent expression with the centrosomal marker γ-tubulin (Fig. 1B). CCHCR1 co-localizes with β-catenin and its phosphorylated form at the centrosome as well (Fig. 1C). Our IEM studies with stable HEK293 Iso1 cells reveal that CCHCR1 is present at the pericentrosomal region at about 200–1000 nm distance apart from the centrioles (Fig. S1D). The CCHCR1 protein is detectable throughout the cell cycle (Fig. 2), partially overlapping with γ-tubulin. The localization is dynamic and fluctuates especially during mitosis. Near the end of cytokinesis, when the dividing cells are still connected, CCHCR1 is also detectable at the midbody (Fig. 2). The co-localization with γ-tubulin was confirmed also in transiently transfected primary normal human epidermal keratinocytes (NHEK) (Fig. S2A).

All four CCHCR1 isoforms are also visible as cytoplasmic granules that do not co-localize with microtubule proteins α- and β-tubulin. A high proportion (75%) of centrosomal proteins contains coiled-coil regions in their structure, as CCHCR1; many of them form cytoplasmic aggregates when overexpressed [29]. Closer microscopic examination of transiently transfected HEK293, HaCaT and NHEK cells reveals that the Iso3Risk construct forms larger granules in the cytoplasm (data not shown). The IEM with transiently transfected COS-7 cells (Fig. S2C) and HaCaT cells (data not shown) support the observation. Immunofluorescent staining with vimentin antibody shows that CCHCR1 granules are not surrounded by a vimentin cage (Fig. S4B) that forms around an aggresome, which is an organelle composed of misfolded aggregated proteins and located adjacent to the centrosome [30]. We also detected that especially in transiently transfected primary keratinocytes (NHEK) the Iso3Risk shows stronger perinuclear staining than the other constructs (Fig. S2B). The staining is not recognized by a cis-golgi marker GM130 (data not shown), which has a function as a regulator of centrosomes as well. As demonstrated in stably transfected HEK293 (Fig. S2D), GM130 surrounds the centrosomal CCHCR1. Otherwise all constructs localize to the centrosome and form cytoplasmic granules also in primary keratinocytes.

Immunofluorescent stainings show that also the endogenous CCHCR1 protein localizes at the centrosome in HEK293 and HaCaT cells (Fig. S3A and B). The expression level is extremely low in both cell lines and in HaCaT cells the small size of centrosomes makes it even more difficult to detect CCHCR1 protein. Unlike the DsRed-tagged CCHCR1 isoforms (tag in the C-terminus), the endogenous protein stained with an antibody against the N-terminal part of isoform 3 is detectable also at the cell-cell borders (Fig. S1A). This suggests a plausible modification or cleavage of the C-terminus, before the transportation of the protein to the cell-cell border. The lower band of CCHCR1 in Western Blot supports the observation and possibility of modification (Fig. S1B). Interestingly, in skin samples the IEM reveals labeling in the close proximity of cell membranes in association with desmosomes both in psoriatic and healthy skin (data not shown).

### CCHCR1 affects cytoskeleton and has a dynamic localization in the cell

**Figure 3:**
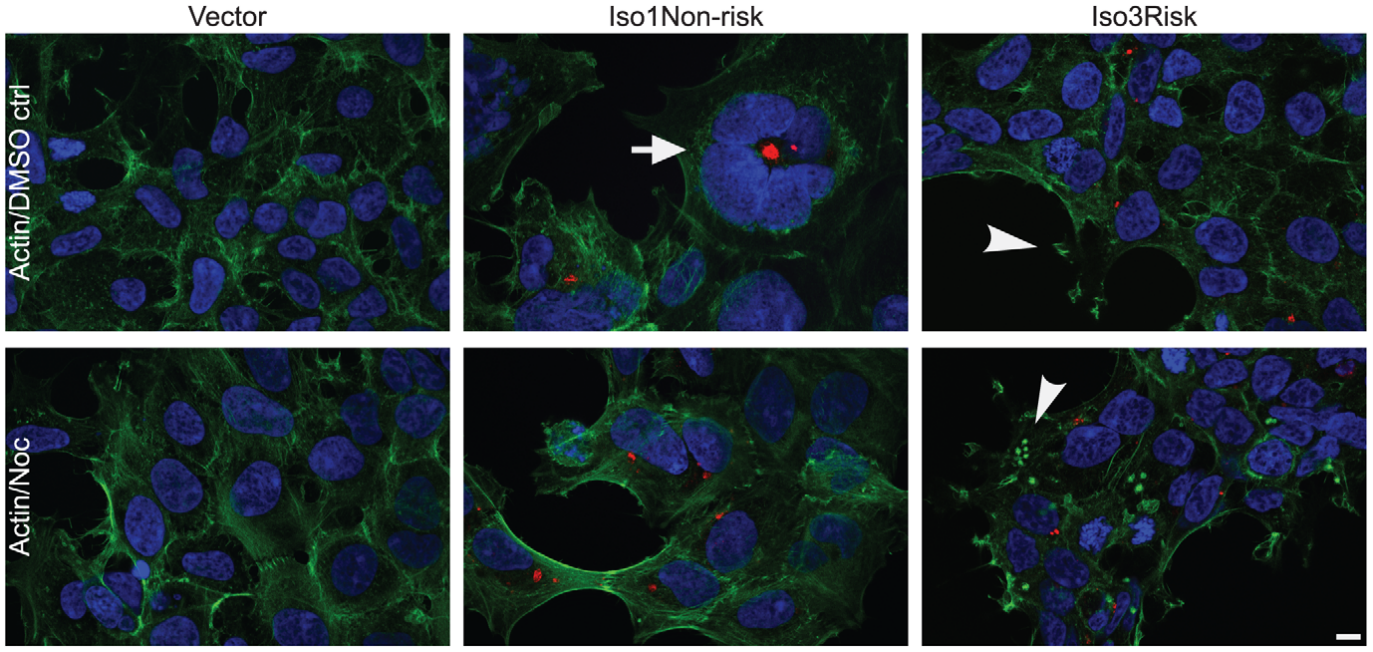
Morphological and cytoskeletal alterations in CCHCR1-overexpressing stable cell lines. Actin staining (green) shows differences in the cell morphology and cytoskeleton between cells expressing different CCHCR1 isoforms (red), here presented vector control, Iso1Non-risk, and Iso3Risk cells. Isoform 1 and 3-expressing cells differ in cell size and shape; Iso1Non-risk cells are larger and rounder than Iso3Risk and their cell nuclei are larger. Furthermore, of the CCHCR1 cell lines especially Iso1Non-risk cells show multilobulated cell nuclei (arrow). The isoform 3-expressing cells also exhibit more pseudopodia with actin rich tips, indicated by an arrowhead (upper panel). The most obvious change in actin organization is observed in the Iso3Risk cells, after treatment with nocodazole (Noc); actin forms punctate staining resembling podosome-like structures, indicated by an arrowhead (lower panel). DAPI stained nuclei are shown in blue. Scale bar 10 µm.

The stable overexpression of CCHCR1 brings out morphological alterations in HEK293 cells; isoforms 1 and 3 have opposite effects on the cell size and shape (Fig. 3; Fig. S4). Iso1Non-risk-expressing cells seem to be bigger in size and rounder in shape, having larger area of cytoplasm than the Iso1Risk cells, whereas both isoform 3-expressing cell lines are even smaller and have more membrane protrusions. Also the size of cell nuclei in interphase differs between isoform 1 and 3 cell lines (*P*<10^−6^) (Fig. S4C). Furthermore, nuclear aberrations such as multilobular nuclei are detectable in the cell lines overexpressing CCHCR1, especially in Iso1Non-risk cells (Fig. 3; Fig. S4A). As the centrosome regulates the organization of microtubules and therefore modulates the cytoskeleton, we studied the relationship between CCHCR1 and the microtubulus network alongside cytoskeletal proteins actin, vimentin, and cytokeratins. We treated the stable cells with nocodazole, an agent able to disrupt microtubule structures. After the treatment, the number of cytoplasmic CCHCR1 granules increases remarkably but also the centrosomal localization is still observable, suggesting a partial dependency of the CCHCR1 localization on microtubules (Fig. S5C). The overexpression of different CCHCR1 isoforms does not have major effects on the microtubulus network but the disruption of the network with nocodazole, however, affected the attachment and shape of the Iso3Risk cells by making them clump together (data not shown).

We studied the effects of CCHCR1 expression on actin (Fig. 3; all CCHCR1 cell lines shown in Fig. S5) and vimentin (Fig. S4) intermediate filaments by immunofluorescence microscopy. The actin cytoskeleton of isoform 1 overexpressing cells is similar to vector control or wild type HEK293 cells. However, phalloidine staining suggests altered actin cytoskeleton of isoform 3 cells (Fig. 3; Fig. S5), which exhibit long filopodial projections with actin-rich tips. The most obvious abnormality is observed after disruption of the microtubulus network with nocodazole; after the treatment, actin is also detectable as punctate staining in the cytoplasm. The actin rich spheres resemble podosomal or invadopodial structures, which are actin rich protrusions of the cell membrane [31]. Based on immunofluorescent staining the CCHCR1 overexpressing cell lines differ slightly also in vimentin organization and expression but immunoblotting shows only minor differences in the protein expression level between different CCHCR1 cell lines (Fig. S4B and D). Disruption of the cytoskeleton with nocodazole, however, lacks similar alterations in vimentin organization as in actin (data not shown).

**Figure 4:**
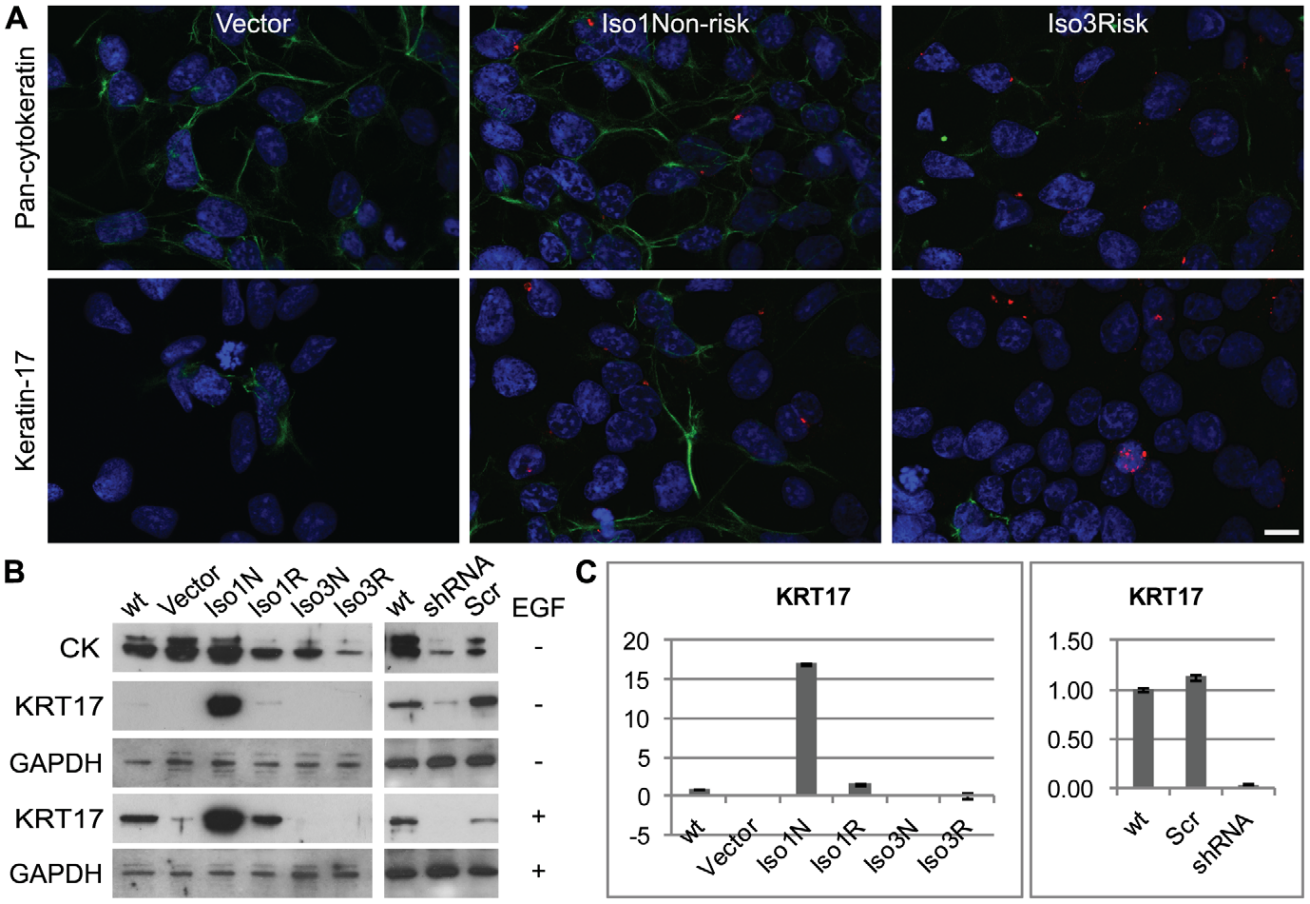
CCHCR1 affects the expression of cytokeratins. (**A, B**) The overall expression of cytokeratins is reduced especially in the Iso3Risk (Iso3R) overexpressing cells shown by (**A**) immunofluorescence staining (here presented vector control, Iso1Non-risk (Iso1N) and Iso3Risk (Iso3R) cells) and (**B**) Western blotting with pan-cytokeratin (CK) antibody (green). The KRT17 expression is increased in the Iso1Non-risk cells (green). The up-regulation of KRT17 is shown by (**A**) immunofluorescence (here presented vector control, Iso1Non-risk and Iso3Risk cells), (**B**) Western Blotting and (**C**) qPCR. (**B**) EGF stimulates KRT17 expression in all the other, except the isoform 3-expressing cells. The silencing of CCHCR1 in HEK293 cells (shRNA) downregulates KRT17 expression, shown by (**B**) Western blotting and (**C**) qPCR. (**B**) The expression of KRT17 under CCHCR1 silencing is not restored by EGF stimulation. (**B**) Also the overall expression of cytokeratins is decreased in CCHCR1 silenced HEK293 cells demonstrated with pan-cytokeratin (CK) antibody. Wt, non-transfected HEK293 cells; shRNA, HEK293 cells transfected with CCHCR1-shRNA construct; Scr, HEK293 transfected with the scrambled sequence. Staining with an antibody for GAPDH was used to control sample loading on SDS-PAGEs. DAPI stained nuclei are shown in blue. Scale bar 10 µm.

Our previous microarray results with transgenic mice suggest that CCHCR1 might regulate the expression of cytokeratins [11]. Immunofluorescent staining of stable CCHCR1 cell lines with a pan-cytokeratin antibody reveals decreased expression in Iso3Risk cells (Fig. 4A, all CCHCR1 cell lines shown in Fig. S6). Similar downregulation of cytokeratin expression in Iso3Risk line is observable by immunoblotting (Fig. 4B). Western blotting reveals also that the silencing of CCHCR1 in HEK293 cells results in an overall reduction of cytokeratin expression (Fig. 4B).

Keratin 17 (KRT17) is implicated in the psoriasis pathogenesis and its expression is altered in experiments with transgenic mice overexpressing CCHCR1 [11], [32]. Immunofluorescent staining of KRT17 in stably transfected CCHCR1 cells reveals increased expression in Iso1Non-risk cells, whereas the labeling in Iso1Risk cells resembles the control cell line (Fig. 4A). In Iso3Non-risk and –Risk cells only a few cells are positive for KRT17. The immunofluorescent results agree well with the qPCR and western blotting (Fig. 4B and C) data showing a significant induction of KRT17 expression in the Iso1Non-risk cells. Furthermore, as shown by immunoblotting and qPCR, the silencing of CCHCR1 downregulates KRT17 expression in HEK293 cells (Fig. 4B and C).

### CCHCR1 regulates EGF-induced STAT3 phosphorylation

**Figure 5:**
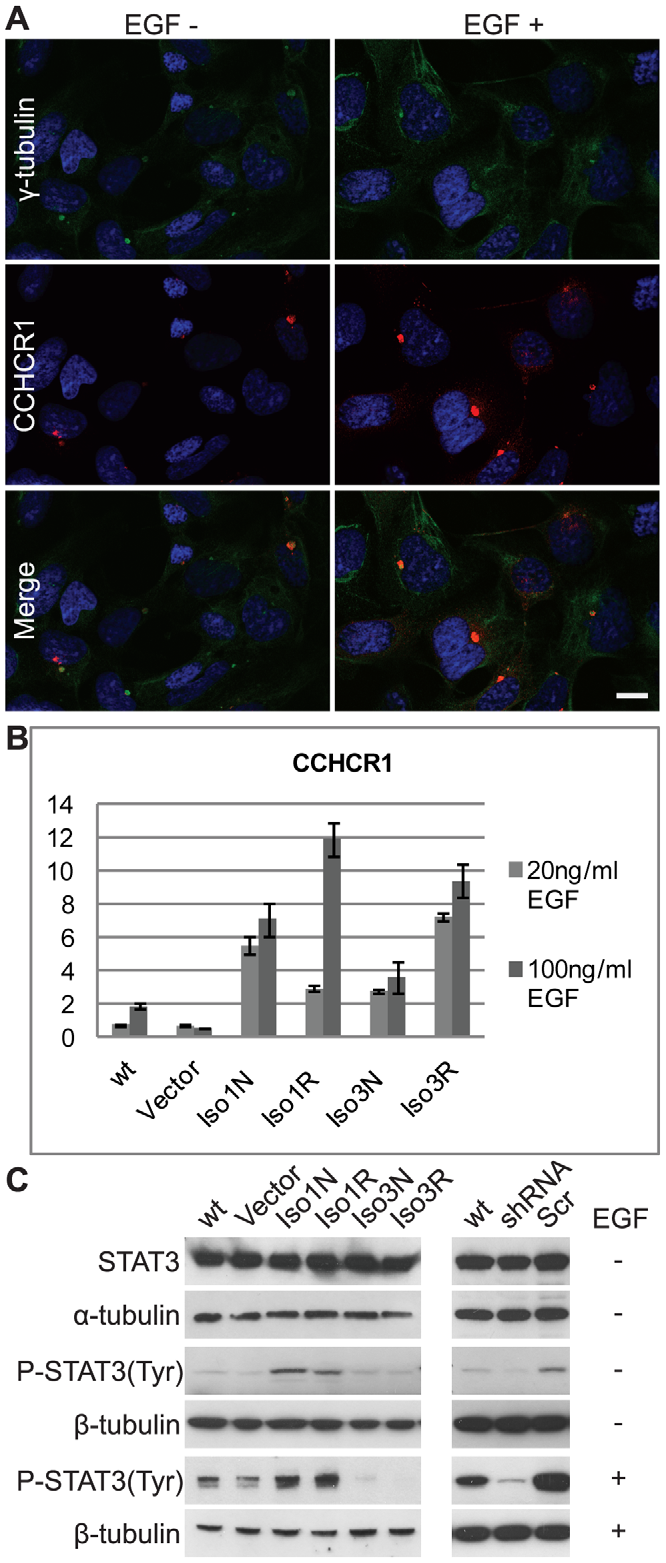
CCHCR1 and EGFR-STAT3 signaling. The localization and expression of CCHCR1 is altered after the stimulation of stable cell lines with EGF as shown by (**A**) immunofluorescence staining and (**B**) qPCR. (**A**) After EGF treatment cytoplasmic CCHCR1 (red) expression is increased though some protein still co-localizes with γ-tubulin (green), thus remains at the centrosome. All isoforms respond similarly to EGF (EGF+), in panel (**A**) Iso1Non-risk cells are shown. Also on RNA level (**B**), when compared with non-treated cells (EGF-), all EGF-treated cell lines show upregulation of CCHCR. Error bars represent SD. Following the EGF treatment (100 ng/ml 18 h) in stably transfected cells, CCHCR1 overexpression has an isoform-specific effect on the STAT3 activation. As shown by Western Blotting (**C**), the EGF-induced Tyrosine 705 phosphorylation of STAT3 is disturbed in both isoform 3-expressing cell lines (Iso3Non-risk (Iso3N) and Iso3Risk (Iso3R)), whereas isoform 1 cells (Iso1Non-risk (Iso1N) and Iso1Risk (Iso1R)) show similar STAT3 phosphorylation as wild type cells (wt). The Iso1Non-risk and in some extent also Iso1Risk cells are able to induce tyrosine 750 phosphorylation without any EGF treatment. The silencing of CCHCR1 in HEK293 cells (shRNA) reduces tyrosine 750 phosphorylation when compared with the control cell line transfected with the scrambled sequence (Scr) or to wild type cells. Overexpression or silencing of CCHCR1 does not affect the expression level of STAT3. Staining with the antibody for β-tubulin was used to control sample loading on SDS-PAGEs. Scale bar 10 µm.

We have recently shown that EGF stimulates CCHCR1 expression in HaCaT keratinocytes [8]. Here, we found that besides inducing CCHCR1 expression the EGF also affects its localization in stably transfected CCHCR1 cell lines (Fig. 5A). After EGF treatment, CCHCR1 is still present at the centrosome but its cytoplasmic localization changes. Furthermore, its expression increases remarkably both on RNA and protein level, shown by qRT-PCR (Fig. 5B) and western blotting (Fig. S1A). All four CCHCR1 isoforms respond similarly to EGF treatment, although the expression level of Iso1Non-risk accelerates the most. We also studied the effects of CCHCR1 isoforms on STAT3 activation by western blotting using antibodies against tyrosine 705 or serine 727 phosphorylated STAT3 and lysine 685 acetylated STAT3. We found that the isoform 1 overexpressing cells show slightly stronger EGF-induced STAT3 tyrosine 705 phosphorylation than the wild type HEK293 cells, whereas the STAT3 phosphorylation is disturbed in isoform 3 cells (Fig. 5C). In addition, silencing of CCHCR1 in HEK293 cells decreases the tyrosine 705 phosphorylation (Fig. 5C). Overexpression or silencing of CCHCR1 affects neither the expression level of STAT3 (Fig. 5) nor its serine 727 phosphorylation or lysine 685 acetylation (Fig. S7). The Iso1Non-risk cells and in some extent also Iso1Risk cells are able to induce tyrosine 750 phosphorylation also in the absence of EGF treatment.

### Effects of CCHCR1 on cell proliferation and apoptosis

**Figure 6:**
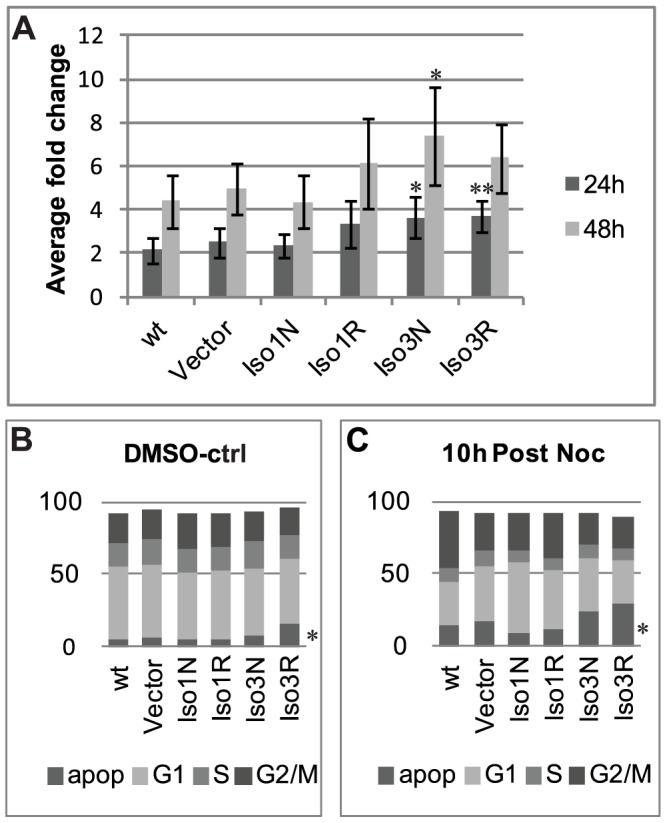
CCHCR1 isoform 3 overexpressing cells Iso3Non-risk and Iso3Risk multiply faster than other stable cell lines. (**A**) Cell proliferation was determined by counting the cell numbers with an automated cell counter (Scepter) during growth periods of 24 or 48 h. Iso1Non-risk (Iso1N) cells show similar proliferation as wild type (wt, HEK293) and vector-control (Vector) cells. The greatest difference is seen between the Iso1Non-risk and isoform 3-expressing cell lines (Iso3N, Iso3R) (*P*<0.003) after 24 h, the latter proliferating significantly faster. (**B**) Cell cycle is similar in all CCHCR1 cell lines shown by percentage values. Higher sub-apoptotic population (apop) in Iso3Risk (Iso3R) cells, however, indicates failure in cell cycle. Cell cycle progression was determined by treating the cells with 0.3 µM nocodazole (Noc) or DMSO-control over night, after which the medium was changed and the cells harvested and fixed at different time points (0 and 10 h shown). DNA was stained with propidium iodine for FACS analysis and the proportion of cells in each of the cell cycle stages (G1, S, G2/M) identified by using the relative DNA content. Asterisks show significant *P*-values calculated with values from the vector-control cell line and the representative cell line (*<0.05>**<0.01).

Nuclear aberrations in stable CCHCR1 cell lines suggest abnormalities in the cell cycle control. Furthermore, isoform 3 cells differ from other cell lines at the growth rate. When the cell number was determined with a fully automated cell counter (Scepter, Millipore), after a culturing of 24 or 48 h, we found that both isoform 3 overexpressing cell lines multiply faster (*P*<0.03 and *P*<0.07, respectively) than the Iso1Non-risk, wild type, or vector control cells (Fig. 6A); after a growth period the increase in the cell number is 40–60% higher in isoform 3 cells than in other cell lines. The Iso1Non-risk cells show similar proliferation as wild type and vector control cells, whereas the growth of Iso1Risk cells resembles isoform 3-expressing cells. The CyQUANT® Cell Proliferation assay (Invitrogen) did not reveal any statistically significant differences but showed a high variation in measurement values (data not shown). Differences in the size of nuclei may have an effect on cell proliferation methods based on DNA staining, such as the CyQUANT® system. The Iso1Non-risk-expressing cells exhibit more multilobular cell nuclei that are able to bind more CyQUANT® reagent than the other cells, especially isoform 3-expressing cells with smaller nuclei. To study the cell cycle, we did FACS assays using propidium iodide staining of the DNA. The cell cycle and progression from synchronized cell phase is otherwise normal in all CCHCR1 cell lines (Fig. 6B), except in Iso3Risk cells, where the population for apoptotic cells is significantly higher (*P*<0.03). Synchronization of cells to the same phase for cell cycle progression measurements was done with 0.3 µM nocodazole over night, after which the media was changed and the cells harvested and fixed at specific time points. FACS demonstrated that after the synchronization the population of apoptotic cells is higher in Iso3Risk line than other cell lines; 30% and 10–24%, respectively (Fig. 6B). The proliferation and cell cycle of CCHCR1-silenced shRNA-cell lines was also measured but lacked significant alterations when compared to control cell lines (data not shown).

## Discussion

### The SNP creating the shorter CCHCR1 isoform associates with psoriasis

The role of *CCHCR1* as a susceptibility gene for psoriasis was strengthened by genome-wide association studies where the SNPs (rs130065, rs130076, rs3130453) from the coding region of *CCHCR1* showed strong association to psoriasis with *P*-values varying between 10^−4^ and 10^−150^ [4], [33], [34], [35]. Even though *HLA-Cw6*, the main marker of *PSORS1*, remains equally or in some studies more strongly associated with psoriasis, the mechanistic support for its role in this disease is missing. In the present study, we cloned a novel longer CCHCR1 isoform 1 resulting from a SNP (rs3130453) that changes a stop codon (*Iso3) into tryptophan (*Iso1). The genetic analyses of psoriasis samples suggests that the *Iso3* allele creating the shorter isoform 3 associates with psoriasis. Here we present that CCHCR1 localizes at the centrosome and via affecting cytoskeletal organization and cell proliferation, has haplotype specific functional consequences relevant to the pathogenesis of psoriasis. Furthermore, our results suggest that CCHCR1 might function in EGFR-STAT3 signaling, previously implicated in psoriasis as well.

### Cellular localization of CCHCR1 suggests a role in processes related to microtubule organization

The expression level of endogenous CCHCR1 protein in cells is extremely low, making its detection difficult. Thus, the exact cellular localization of the CCHCR1 protein was unknown hitherto. Here, we show with immunofluorescent staining and DsRed-tagged protein constructs that both CCHCR1 isoforms 1 and 3 colocalize with γ-tubulin at the centrosome. This is supported by previous mass spectrometry studies, where CCHCR1 was detected from extracted centrosomes [29], [36]. Interestingly, we also demonstrated that CCHCR1 co-localizes at the centrosome with β-catenin, a protein implicated in psoriasis [28]. The centrosome has a crucial function in mitosis. Numerous centrosomal proteins play a role in cytokinesis as well and are identifiable at the intercellular bridge connecting the dividing cells. These including γ-tubulin and β-catenin [24], [37], [38]. During cytokinesis, CCHCR1 is also visible at the midbody between daughter cells. In addition, the formation of multilobulated nuclei in CCHCR1 overexpressing cell lines suggests a role in cell division and cytokinesis. In skin sections the IEM reveals CCHCR1 expression also in the proximity of cell membrane and desmosomes. This may be related to the role of desmosomes in skin; during epidermal differentiation desmosomes replace centrosomes as organizators and regulators of microtubulus cytoskeleton [39].

### CCHCR1 regulates cytoskeletal organization

The stably transfected cells overexpressing CCHCR1 show isoform- and haplotype-specific morphological changes in cell size and shape, suggesting abnormalities in the organization of cytoskeleton. The centrosome regulates the organization of microtubules and through its influence on the cytoskeleton it affects cell shape and size [21]. Our previous microarray expression data from transgenic CCHCR1 mice supported a role in cytoskeleton organization [11]. Here, we demonstrate that CCHCR1 affects the arrangement and expression of actin, vimentin, and cytokeratins; Iso3Risk cells show aberrant actin and vimentin skeleton organization and downregulation of vimentin and cytokeratins. In psoriatic skin, the expression levels and patterns of various cytokeratins are dramatically altered and the amount of vimentin mRNA is reduced [40], [41]. Interestingly, vimentin is involved in the cell proliferation and maintenance of cell shape [42], [43], [44], which are biological processes affected also by CCHCR1. Nocodazole is an agent that disturbs the formation of microtubule filaments and by this action suggested to alter actin cytoskeleton [45]. In stable CCHCR1 cell lines, the effect on actin cytoskeleton is the most obvious in cells treated with nocodazole. Especially in isoform 3-expressing cells, in addition to filaments, actin forms clusters or individual spot-like structures resembling podosomes or invadopodia, which are actin-containing structures involved in cell migration and invasion. Structures of this type are referred to as invadopodia when they are found in cancer cells and as podosomes when found in normal cells. As the formation of podosomes is suggested to be microtubule dependent [31], the formation of the structures in CCHCR1-expressing cells in consequence of microtubule disruption implies invadopodial nature. Interestingly, several diseases are associated with impairment of podosome formation, most notably Wiskott–Aldrich syndrome, an X-linked recessive disease with eczema, thrombocytopenia and severe immune deficiencies [31]. Vimentin is present in the elongated mature invadopodia [46] but similar spot-like pattern formation after the nocodazole treatment, as with actin, was not observable with vimentin in cells overexpressing CCHCR1. Nocodazole also affected CCHCR1 expression and localization in stable cell lines, suggesting that microtubules regulate CCHCR1. After the treatment, CCHCR1 is still partly in association with the centrosomes, but also dispersed into the cytoplasm as larger aggregates. These aggregates, however, do not colocalize with the actin-rich spots.

### CCHCR1 affects the expression of keratin 17, a hallmark for psoriasis

The overall expression of cytokeratins decreases most evidently in Iso3Risk cells and in some extent also in Iso3Non-risk and Iso1Risk cells. This agrees with our results from experiments with transgenic mice that overexpress CCHCR1 isoform 3 with the *WWCC risk-allele, demonstrating keratins as the most strongly downregulated gene group when compared with non-risk allele mice [11]. Here we show that in HEK293 cells, the overexpression of CCHCR1 isoform 1 with the non-risk allele upregulates the expression of keratin 17 (KRT17), a hallmark and plausible auto-antigen for psoriasis [32], [40], [47], [48].Correspondingly, the silencing of CCHCR1 in HEK293 cells, which are homotsygous for the *Iso1* allele and are therefore able to express also the isoform 1, reduces the KRT17 expression. The expression in isoform 3 overexpressing cells is also decreased, which is in contrast to the effects seen in isoform 1 overexpressing cells. The microarray results from the skin of transgenic mice [11], however, suggested that KRT17 expression is increased in mice expressing isoform 3 with the risk-allele (CCHCR1*Iso3WWCC). In human healthy epidermis, KRT17 is absent but overexpressed in psoriatic lesions [32], [40], [49], where it is suggested to promote epithelial proliferation, modulate immune responses, and to have antiapoptotic effects [50], [51]. The opposite KRT17 expression results obtained with the overexpressing CCHCR1 cell lines and transgenic mice might be explained by different expression profiles of keratins between cultured epithelial cells, like HEK293, and skin. The environment and cellular interactions are different in cells cultured in monolayers and cells in intact tissues. In addition to the allele-specific effects, the expression level of CCHCR1 may affect its downstream signaling; effects of isoforms 1 and 3 on KRT17 expression may also be dependent on their amount in cells. Notably, KRT17 is also known to influence cell growth and size [52] by promoting protein synthesis. Thus, the upregulation of KRT17 in Iso1Non-risk cells may partially explain the increased cell size in this cell line.

### CCHCR1 regulates EGF-induced STAT3 activation

Our results implicate that CCHCR1 may function in EGFR-STAT3 signaling pathway. This is based on our observations that EGF influences CCHCR1 expression [8], and in turn CCHCR1 regulates EGF-induced STAT3 activation. The EGF treatment increases the CCHCR1 expression both on mRNA and protein level also in stably transfected CCHCR1 cell lines. EGF is known to stimulate the expression of several genes through post-transcriptional mechanisms, genes, such as β-catenin and thrombospondin-1 [53], [54]. We suggest that the EGF-induced increase in CCHCR1 expression is based both on mRNA and protein stabilization. Here, we show that CCHCR1 regulates EGF-induced STAT3 activation in an isoform-specific manner: STAT3 tyrosine 705 phosphorylation is disturbed in the isoform 3-expressing cells, whereas the isoform 1 cells activate STAT3 phosphorylation slightly more than the wild type cells. Correspondingly, the silencing of CCHCR1 in HEK293 cells (homotsygous for *Iso1WWCC) decrease the phosphorylation. This is a relevant observation to the function of CCHCR1 even though the CCHCR1*Iso3WWCC haplotype associates with decreased STAT3 activation, while epidermal keratinocytes within psoriatic skin are characterized by activated STAT3, and in transgenic mice the expression of constitutively active Stat3 leads to a psoriasis like skin phenotype [20], [55]. However, the regulation of STAT3 phosphorylation in skin is probably much more complicated than in cells cultured in a monolayer, and in psoriatic skin in addition to EGF numerous other cytokines play a role. Overexpression of CCHCR1 isoforms does not affect the expression level of STAT3 or its serine 727 phosphorylation or lysine 685 acetylation. Tyrosine 705 phosphorylation is believed to be crucial for STAT3 activation and nuclear translocation [19], [56]. Constitutive activation of STAT3 in psoriatic skin may result from the persistent stimulation of EGFR as its multiple ligands are found as increased levels in psoriatic skin [15], [16], [20] and as a tyrosine kinase receptor, EGFR is able to phosphorylate STAT3 directly or indirectly without Janus kinase (JAK) [17], [19], [20]. STAT3 regulates various processes in skin, including cell proliferation, differentiation, and apoptosis. The regulation is mediated through the transcription of genes such as cyclin-D, MYC, and BCL-2 [19], [20]. Interestingly, upregulation of *KRT17* expression is also mediated by STAT3 [49], suggesting regulation of KRT17 induction in CCHCR1 isoform 1-expressing HEK293 cells through this pathway. STAT3 is also known to control processes without affecting transcription, for instance it regulates centrosome doubling by modulating γ-tubulin levels [57]. Furthermore, it regulates the depolymerization of microtubules by interacting with stathmin, a tubulin binding protein [58].

### CCHCR1 isoform 3 affects cell proliferation

Our earlier findings implicated a role for CCHCR1 in keratinocyte proliferation [10]. Here we show that the overexpression of the novel longer CCHCR1 isoform 1 lacks significant effects on cell proliferation or cell cycle progression in stable HEK293 cell lines. Isoform 3 overexpressing cells, however, multiply faster and the Iso3Risk cells also show increased apoptosis. After nocodazole synchronization the rate of apoptosis increases even further as the cell cycle progresses, suggesting failure to proceed from cell cycle check points, which may result from the cytoskeletal aberrations. We have earlier shown that overexpression of CCHCR1 isoform 3 affects cell proliferation in the skin of transgenic mice; the number of proliferative cells was 20% lower in mice with the CCHCR1*WWCC risk allele than in non-risk allele mice. In stable cell lines a similar trend (14%) is observable between Iso3Non-risk and -Risk cell lines (*P*>0.05). A more significant difference in cell proliferation, however, is between isoform 3-expressing cell lines and Iso1Non-risk cells; both the Iso3Non-risk and –Risk cells multiply faster than the Iso1Non-risk, wild type or vector control cells. The proliferation results were not validated by the DNA-based CyQUANT® system, due to differences in the size of the nuclei between the cell lines. In transgenic mice the various inductions and the expression level of CCHCR1 may have affected to the proliferation results [11]. There may be also differences in CCHCR1 function between mouse and human as well as between cell types.

## Conclusions

Functions presented here for CCHCR1 in cytoskeleton organization and cell proliferation are overlapping and mediated through centrosomes. In addition to the regulation of cell cycle and cytokinesis, centrosomal proteins can regulate diverse other microtubule-based processes such as vesicle docking and mitochondria transporting [21], [59], [60]. Centrosomal proteins can be very dynamic in trafficking between the centrosome-bound and cytoplasmic pool, interacting with numerous proteins. CCHCR1 also shows localization in various other compartments in the cell; midbody, cytoplasm, and in the proximity of the cell membrane and desmosomes. It enhances the synthesis of steroids by interacting with the mitochondrial steroidogenic acute regulatory protein (StAR) [8] and here we propose that it may indirectly induce steroidogenesis as well. CCHCR1 regulates cytoskeleton, including vimentin that also plays a role in the synthesis of steroids by modulating the motility of mitochondria and by binding cholesterol [61], [62]. In addition, CCHCR1 interacts with the RNA polymerase II subunit 3 (RPB3) [63] and controls its localization. RPB3 regulates the expression and compartmentalization of vimentin through the action of eukaryotic translation elongator factor 1 γ (eEF1γ) [64], [65].Therefore,the effect of CCHCR1 on vimentin organisation could be mediated through its interaction partner RPB3. The inhibitory effect of CCHCR1 isoform 3 on tyrosine phophorylation of STAT3 may also arise from cytoskeletal alterations caused by CCHCR1. STAT3 is known to interact with cytoskeletal structures and drugs used in cancer treatment, such as microtubule stabilizer paclitaxel and microtubule inhibitor vinorelbine that decrease the tyrosine phosphorylation of STAT3 and thus inhibit the expression of STAT3 target genes [66]. Paclitaxel is also known to decrease the association between STAT3 and microtubules. Taken together; we suggest that, as a dynamic centrosomal protein, CCHCR1 is able to interact with several proteins and modulate multiple cellular processes that are involved in psoriatic skin. We also show that the N-terminal part of the isoform 1 affects the function of CCHCR1 both independently and in combination with the non-risk/risk (*RRGS/WWCC) status. Most importantly, we show that the SNP (rs3130453) controlling the translation start site associates with psoriasis, is in high linkage disequilibrium with the psoriasis risk-haplotype SNP rs130076, and that CCHCR1has allele-specific consequences on pathways and functions relevant to the pathogenesis of the disease.

## Materials and Methods

### Patient material

In genotyping we utilized DNA samples that have been used in earlier studies. The Swedish and Finnish psoriasis family material for genotyping was recruited as previously described: the Swedish blood samples were collected with the help of the Swedish Psoriasis Association and approved by the Regional Ethics Committee [67], the Finnish samples were approved by the Ethics Committees of Helsinki, Turku, Tampere, and Oulu University Central Hospitals and Central Hospital of Päijät-Häme [68], [69]. The skin samples for expression studies were dermatome biopsies measuring 5×2 cm, 4–6/1000 inches thick (Zimmer® dermatome, Minneapolis, MN). Psoriasis patients were sampled from both the lesional and non-lesional skin (n = 5). Healthy skin (n = 2) was obtained from reductive mammoplastic or microvascular free flap surgery patients. The collection of skin samples was approved by the Ethics Committee of the Hospital District of Helsinki and Uusimaa and by the Committee of Skin and Allergy Hospital, Helsinki University Central Hospital. All participants gave written informed consent for the blood and skin sample collections and the studies followed the Declaration of Helsinki Guidelines.

### SNP genotyping and association analysis

The samples used for the genotyping consisted of trios from 508 psoriasis families in total, including 245 Finnish and 263 Swedish families. We genotyped the SNPs rs3130453 and rs130076 by using DNA extracted from the Finnish and Swedish family samples. Genotyping was done using TaqMan allelic discrimination assays (C_2452221_10 and C_2452225_10, respectively) with probes and primers designed and synthesized by the supplier (Applied Biosystems). Transmission disequilibrium test (TDT) was performed using HaploView [70]. To correct for multiple testing, we performed permutation tests (100000 permutations).

### Cloning and constructs

Cloning of cDNAs encoding the isoform 3 with the risk or non-risk haplotype (SNPs rs130065, rs130076, rs130079, rs1576) has been described previously [3], [11]. The cDNA corresponding for the N-terminal part of the isoform 1 was obtained from HaCaT RNA by reverse transcription-PCR (RT-PCR) using primers (Table S2) designed according to the reference sequence NM_001105564.1. The isoform 1 non-risk and risk constructs were engineered combining the novel N-terminal part to the previous isoform 3 non-risk or risk cDNAs using *XbaI* site and ligation. All cDNAs were prepared into the pCMV5 and pDsRed-Monomer-N1 (Clontech) vectors. To prepare short hairpin shRNA constructs for CCHCR1 silencing complementary oligos (Table S2) based on previous experiments [7] were annealed and cloned into the pRNAT-CMV3.2/Neo vector with a GFP tag (GenScript) using *BamHI* and *XhoI* sites. Oligos were designed with GenScript program and scrambled sequence was used to prepare a control construct.

### Cell cultures and transfections

HEK293 cells (American Type Culture Collection) were grown in Dulbecco's Modified Eagle's medium (DMEM) supplemented with 10% fetal calf serum (FCS) and penicillin-streptomycin (P/S). HaCaT cells [71] were kindly provided by Prof. N.E. Fusenig (Heidelberg, Germany) and grown in DMEM supplemented with 5% FCS and P/S. Normal Human Epidermal Keratinocytes (NHEK, PromoCell) were grown in Keratinocyte Growth Medium 2 (PromoCell) supplemented with 0.06 mM calcium and P/S. Plasmid transfections were performed with FuGENE HD reagent (Roche Diagnostics) according to the manufacturers' instructions. The cover slips or cell culture wells were coated with Rat Tail Collagen I (Gibco, Invitrogen) when used for immunofluorescent stainings or nocodazole treatment of HEK293 and NHEK cells.

### Generation of stable cell lines and cell treatments

To generate overexpressing cell lines CCHCR1-pDsRed-constructs or vector were transfected into HEK293 cells using standard methods. Cells were grown in the DMEM culture medium containing 400 µg/ml G418 antibiotics (Roche). After 10–14 days of selection resistant colonies were picked and screened for CCHCR1 expression by quantitative PCR (qPCR) and fluorescence microscopy. Protein expression of selected clones was verified by western blotting and immunofluorescence staining [3], [10]. Stable shRNA cell lines were generated in HEK293 cells using the same transfection and selection protocol as with overexpressing cell lines. After G418 selection resistant clones were screened for plasmid integration by fluorescence microscopy and for CCHCR1 silencing by qPCR. For the treatment with epidermal growth factor (EGF) subconfluent cells were grown in the presence of 20 ng/ml or 100 ng/ml EGF (Sigma). After 2, 6, or 18 h cells were lysed for western blotting or RNA isolation. Experiments were repeated three times (western blotting) or included triplicate samples (qPCR). Disruption of microtubulus network was performed by incubating the HEK293 stable cells one hour with 1 µM nocodazole at 37°C. The cell cycle was synchronized by incubating the cells with 0.3 µM nocodazole over night at 37°C. DMSO was used as a control.

### Antibodies

Antibodies in various immunostainings were: mouse monoclonals against actin (A3853, Sigma); β-catenin (#610154, Transduction Laboratories); cis-golgi marker GM130 (#610822, BD Transduction Laboratories); keratin 17 (2D4-1G9, Sigma); pan-cytokeratin (sc-58828, Santa Cruz); Stat3 (#9139, Cell Signaling Technology); P-Stat3 Ser727 (#9136, Cell Signaling Technology); acetyl-Stat3 Lys685 (#2523, Cell Signaling Technology); α-, β- and γ-tubulins (T5192, T5201, T5326, Sigma), and vimentin (V6389, Sigma). Rabbit antibodies used in the study were against: CCHCR1 [3]; P(S33/37/T41)-β-catenin (#9561, Cell Signaling technology); and P-Stat3 Tyr705 (# 9145, Cell Signaling Technology). Antibodies produced in goat were against CCHCR1 (sc-66231, Santa Cruz Biotechnology); and glyceraldehyde-3-phosphate dehydrogenase (GAPDH) (sc-20357, Santa Cruz Biotechnology).

### Immunofluorescence

For immunofluorescence studies, cells were fixed with methanol for 5 min at −20°C or with 4% paraformaldehyde-phosphate buffered saline (PBS) solution for 15 min at room temperature. After paraformaldehyde fixation cells were permeabilized with 0.1% Triton-X100 -PBS. For colocalization stainings of endogenous CCHCR1, the HEK293 and HaCaT cells were incubated over night at 4°C with the antibodies against CCHCR1 and γ-tubulin or phospho-β-catenin (only HEK293 cells). Stable cell lines expressing pDsRed tagged CCHCR1 isoforms were incubated one hour at room temperature with the antibodies against following proteins: γ- or α-tubulin, β-catenin, P(S33/37/T41)-β-catenin, vimentin, GM130, KRT17, pan-cytokeratins and vimentin. Actin was detected using the Alexa Fluor 488 conjugated phallodin (Invitrogen, Molecular Probes). NHEK cells transfected with CCHCR1 constructs were stained with rabbit antibodies for CCHCR1 one hour and mouse antibodies for γ-tubulin one hour. Alexa Fluor 555 and 488 conjugated IgGs (Invitrogen, Molecular Probes) were used as secondary antibodies. The nuclei were stained with DAPI and the pictures taken with Zeiss LSM 5 Duo confocal microscope.

### Immunoelectron microscopy

For IEM, transfected cells were fixed for one hour with 4% paraformaldehyde-PBS solution, immersed in 12% gelatin-PBS solution, followed by immersion in 2.3 M sucrose-PBS solution. Skin biopsies were fixed with paraformaldehyde from 6 to 12 h followed by immersion in sucrose-PBS solution. Samples were frozen in liquid nitrogen, and thin cryosections were cut with a Leica Ultracut UCT microtome. After which they were labeled with antibodies against CCHCR1 followed by incubation with gold conjugated protein A. Labeling was detected with a Philips CM100 transmission electron microscope.

### RT-PCR and Quantitative real-Time PCR

Tissue samples for RNA isolation were immediately immersed into RNAlater Solution (Ambion) and stored according to manufacturer's instructions. RNA extraction from cell lines was done with RNeasy Plus Mini kit (Qiagen) and from tissue (skin dermatome) samples with miRNeasy Mini kit (Qiagen) complemented with DNase treatment (RNase-Free DNase Set, Qiagen) according to manufacturer's instructions. Total RNA was reverse transcribed to cDNA using TaqMan Reverse Transcription Reagents (Applied Biosystems) or Transcriptor High Fidelity cDNA Synthesis Kit (Roche Applied Science). The expression of *CCHCR1* transcript variants 1 and 3 in different tissues and cell lines was studied with RT-PCR from human Multiple Tissue cDNA and Fetal and Tumor panels (Clontech), HaCaT and HEK293 cell lines, and normal human epidermal keratinocytes (NHEK) using variant specific primers (Table S2). PCR products were analyzed by agarose gel electrophoresis. Primers for glyceraldehyde-3-phosphate dehydrogenase (*GAPDH*) expression were used to control amplification. *CCHCR1* expression was measured from cell lines and skin biopsies using qPCR (7500 Fast Real-Time PCR System, Applied Biosystems) either with TaqMan reagents (TaqMan Universal PCR Master Mix, pre-designed systems for CCHCR1 #Hs00219401_m1 and GAPDH #4310884E, Applied Biosystems) or SYBR green reagents (SYBR Green PCR Master Mix, Applied Biosystems) and *CCHCR1* specific primers (Table S2). Pre-designed TaqMan system (Hs01588578_m17, Applied Biosystems) was used to study the *KRT17* expression. TATA binding protein (*TBP*) and *GAPDH* were used as control genes in quantification [72].

### Western blotting

Cells grown on 6-well plates were homogenized into 300 µl of Laemmli buffer containing 5% β-mercaptoethanol. A total of 15 ug or 30 ug of protein per sample was analyzed by SDS-PAGE using 7.5% polyacrylamide gels. Western blot analysis was carried out by standard protocols using the following primary antibodies against: actin, CCHCR1, KRT17, pan-cytokeratin Stat3, P-Stat3 Tyr705, P-Stat3 Ser727, acetyl-Stat3 Lys685, and vimentin. Immunostaining with antibodies against β-tubulin or GAPDH were used to control loading. After incubation with the appropriate horseradish peroxidase-conjugated secondary antibodies (Sigma), signals were detected with an enhanced-chemiluminescence (ECL) substrate (Amersham Biosciences/GE Healthcare).

### Flow cytometry and cell proliferation measurements

To study cell cycle in stable CCHCR1 cell lines by flow cytometry, cells grown as duplicates on 12-well plates were trypsinized, pooled, and fixed in ice cold 70% ethanol and stored at −20°C over night. To determine cell cycle progression after synchronization, the cells were synchronized into G2/M-phase by treating the cells with 0.3 µM nocodazole over night (DMSO was used as a control). The media were changed and the cells collected and fixed at different time points. The cells were divided into duplicate samples on 96-well plates and then washed two times with PBS. After treating the cells with 0.1 mg/ml ribonuclease A in PBS for 30 min at 37°C, the nuclei were stained with 0.025 mg/ml propidium iodide (PI) solution (P1304MP, Invitrogen) in 2% FCS-PBS. FACSarray (Becton Dickinson) was used for the detection of PI staining and cell cycle analysis. The experiment was repeated three times. The cell proliferation of stable CCHCR1 cell lines was determined with the automated cell counter (Scepter, Millipore). Cells were seeded at the density of 2.5×10^5^ on 12-well plates and after 24 and 48 h of incubation at 37°C they were trypsinized and counted. The experiment was repeated six times. We also utilized the CyQUANT® Cell Proliferation assay (Invitrogen) to study cell proliferation. Briefly, the cells were seeded at the density of 0.5×10^4^ or 1.0×10^4^ on 96-well plates coated with Rat Tail Collagen I (Gibco, Invitrogen) and allowed to grow at 37°C for 0–48 h. The medium was discarded at specific time points and the cells frozen and stored in −80°C for analysis. The staining was performed according to manufacturer's protocol and the fluorescence measured with Victor Multilabel reader (Wallac, excitation: 480 nm, emission: 530 nm). The size of the nuclei in the stable cell lines was measured from the confocal microscope pictures in which the nuclei were stained with DAPI, using the Zeiss LSM Image Browser 4.2 (Carl Zeiss MicroImaging GmbH).

### Statistical analysis

All statistical analyses were made using Microsoft Office Excel. Statistical comparisons between data sets were made with Student's t-test and *P*<0.05 was considered significant.

## Supporting Information

Figure S1 **Establishment of stable cell lines overexpressing CCHCR1.** CCHCR1 expression in stable cell lines was verified by (**A**) immunofluorescence staining and (**B**) Western blotting. (**A**) As shown by immunofluorescence, all DsRed-tagged CCHCR1 isoforms (red) are recognized by the CCHCR1 antibody (green). In contrast to the C-terminally tagged DsRed-CCHCR1, the N-terminally targeted CCHCR1 antibody detects endogenous protein also at the cell-cell borders (white arrows). DAPI stained nuclei are shown in blue. Scale bar 10 µm. (**B**) Western blotting demonstrates CCHCR1 expression in all four cell lines: Iso1Non-risk (Iso1N), Iso1Risk (Iso1R), Iso3Non-risk (Iso1N) and Iso3Risk (Iso1R) and, in reducing conditions, the antibody against CCHCR1 detects polypeptides of expected size. Interestingly, the expression of Iso3Non-risk isoform is higher than other CCHCR1 isoforms, although the CCHCR1 RNA expression in Iso3Non-risk cells is rather similar to other cell lines (a longer exposure shown in the right-side panel). The cells were grown also in the presence of EGF, which increases the protein level. (**C**) The IEM studies of stably transected HEK293 cell lines demonstrate that CCHCR1 is present at the pericentrosomal region at about 200–1000 nm distance apart from the centrioles. Scale bar 500 nm (**D**) Stable shRNA-expressing cell lines were verified by qPCR. The silencing of CCHCR1 in HEK293 cells (shRNA) downregulates its expression by 50–60%. Scrambled sequence was used to generate control cell line (Scr).
(TIF)

Figure S2 **Expression pattern of CCHCR1 in transfected cells.** (**A**) CCHCR1 (red) colocalizes with γ-tubulin (green) also in transiently transfected primary keratinocytes. (**B**) The Iso3Risk shows stronger perinuclear staining than the other constructs. Immunofluorescence staining of transfected cells with CCHCR1 antibody suggests that the cytoplasmic granules are smaller when formed by the Iso3Non-risk or the Iso1 constructs than by the Iso3Risk construct. (**C**) IEM of transfected COS-7 supports the observation; the gold label in the cytoplasm is observed as clusters that are larger in the Iso3Risk than Iso3Non-risk cells. (**D**) Cis-golgi marker GM130 (green) surrounds and has partial colocalization with the centrosomal CCHCR1 (red) in stably transfected CCHCR1 cell lines (here shown Iso1Non-risk). DAPI stained nuclei are shown in blue. Scale bar 10 µm.
(TIF)

Figure S3 **Localization of endogenous CCHCR1.** Immunofluorescence staining shows that the endogenous CCHCR1 localizes at the centrosome in (**A**) HEK293 and (**B**) HaCaT cells. Cells were stained with CCHCR1 (green) and γ-tubulin (red) antibodies. The last picture shows magnification of the centrosomal region, marked in the merge channel pictures. DAPI stained nuclei are shown in blue. The scale bars are 10 µm and 2 µm (the magnified picture).
(TIF)

Figure S4 **Morphological and cytoskeletal differences in stably transfected CCHCR1 cell lines.** Comparison of all four CCHCR1 and control cell lines stained with antibodies against (**A**) α-tubulin and (**B**) vimentin. The stainings (green) reveal alterations in the cell morphology and cytoskeleton of cells expressing different CCHCR1 isoforms (red). (**A**) The α-tubulin staining pattern is otherwise unchanged in CCHCR1 overexpressing cells. Especially the Iso1Non-risk cell line exhibits multinuclei (indicated with an arrow). (**C**) The average size of the nuclei is significantly smaller in the isoform 3-expressing cells when compared to the vector-control cell line and even more significant when compared to Iso1Non-risk cells. Asterisks show significant *P*-values calculated with values from the vector-control cell line and the representative cell line (*<0.05>**<0.01>***<0.001). (**B**) Vimentin expression and organization is disturbed in both Iso3Non-risk and -risk cell lines as shown by immunofluorescence. (**D**) Immunoblotting with vimentin antibody, however, lacks evidence for major changes in expression between stable CCHCR1 cell lines. A magnification of the centrosomal region from Iso1Non-risk cells with the vimentin staining (**B**) shows that the CCHCR1 is not surrounded by a vimentin cage, which is typical for aggresomes. DAPI stained nuclei are shown in blue. Scale bar 10 µm.
(TIF)

Figure S5 **Microtubule disturption shows altered organization of the actin cytoskeleton in CCHCR1 Iso3Risk overexpressing cells.** (**A**) Phalloidine staining shows that isoform 3-expressing cells exhibit more pseudopodia with actin rich tips, indicated by an arrowhead (Iso3Non-risk, Ctrl). (**B**) The most obvious change in actin organization is observed in the Iso3Risk cells, after treatment with nocodazole (Noc); actin forms punctate staining resembling podosome-like structures, indicated by an arrowhead (Iso3Risk, Noc). (**C**) The localization of CCHCR1 is partially changed after the disruption of microtubule network with nozodazole. DAPI stained nuclei are shown in blue. Scale bar 10 µm.
(TIF)

Figure S6 **CCHCR1 affects the expression of cytokeratins.** Immunofluorescence staining of (**A**) pan-cytokeratin and (**B**) keratin 17 in all four CCHCR1 overexpressing cell lines and vector control cells. (**A**) The overall expression of cytokeratins is reduced especially in the Iso3Risk-expressing cells. (**B**) Iso1Non-risk-expressing cells show an increase in the expression of keratin 17. DAPI stained nuclei are shown in blue. Scale bar 10 µm.
(TIF)

Figure S7 **Overexpression of CCHCR1 in stable cell lines does not affect STAT3 serine 727 phosphorylation or lysine 685 acetylation.** Staining with an antibody for β-tubulin or GAPDH was used to control sample loading on SDS-PAGEs.
(TIF)

Table S1 **Expression of CCHCR1 transcript variants 1 and 3 in various human tissues and cell lines.** Transcripts did not show major differences in their expression; both were detectable in all tissues and cells studied. Here shown band intensities of RT-PCR samples run on agarose gel: from low (+) to high, (+++++).
(XLS)

Table S2 **Sequences of primers used in the present study.**
(XLSX)
