# Supplementary figures and images for: Centrosomal Localization of the Psoriasis Candidate Gene Product, CCHCR1, Supports a Role in Cytoskeletal Organization
Source: PLoS One. 2012 Nov 26;7(11):e49920. doi: 10.1371/journal.pone.0049920 (PMC3506594; doi:10.1371/journal.pone.0049920)

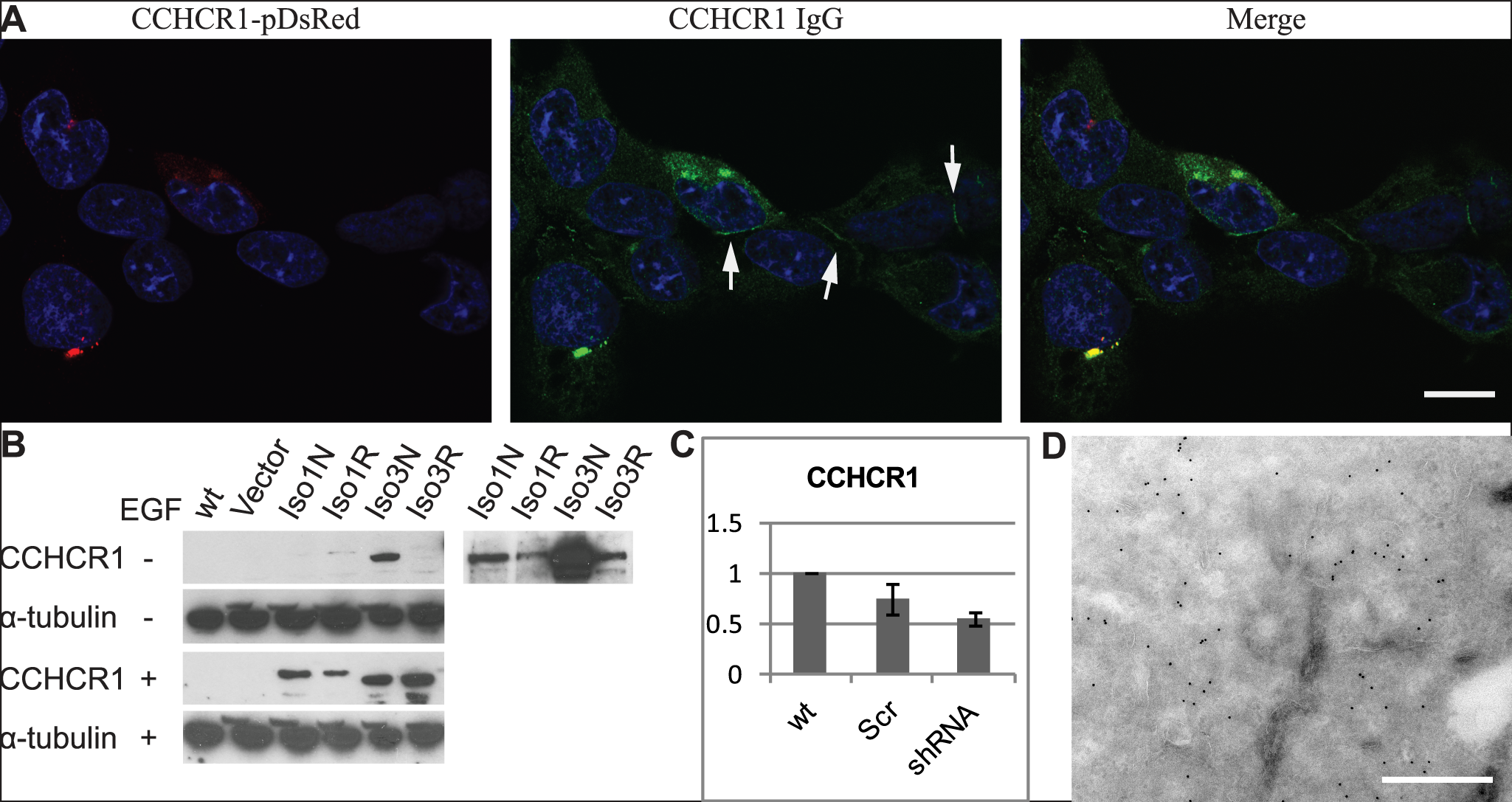

Supplement: Figure S1 — Establishment of stable cell lines overexpressing CCHCR1. CCHCR1 expression in stable cell lines was verified by (A) immunofluorescence staining and (B) Western blotting. (A) As shown by immunofluorescence, all DsRed-tagged CCHCR1 isoforms (red) are recognized by the CCHCR1 antibody (green). In contrast to the C-terminally tagged DsRed-CCHCR1, the N-terminally targeted CCHCR1 antibody detects endogenous protein also at the cell-cell borders (white arrows). DAPI stained nuclei are shown in blue. Scale bar 10 µm. (B) Western blotting demonstrates CCHCR1 expression in all four cell lines: Iso1Non-risk (Iso1N), Iso1Risk (Iso1R), Iso3Non-risk (Iso1N) and Iso3Risk (Iso1R) and, in reducing conditions, the antibody against CCHCR1 detects polypeptides of expected size. Interestingly, the expression of Iso3Non-risk isoform is higher than other CCHCR1 isoforms, although the CCHCR1 RNA expression in Iso3Non-risk cells is rather similar to other cell lines (a longer exposure shown in the right-side panel). The cells were grown also in the presence of EGF, which increases the protein level. (C) The IEM studies of stably transected HEK293 cell lines demonstrate that CCHCR1 is present at the pericentrosomal region at about 200–1000 nm distance apart from the centrioles. Scale bar 500 nm (D) Stable shRNA-expressing cell lines were verified by qPCR. The silencing of CCHCR1 in HEK293 cells (shRNA) downregulates its expression by 50–60%. Scrambled sequence was used to generate control cell line (Scr). (TIF) [file pone.0049920.s001.tif]

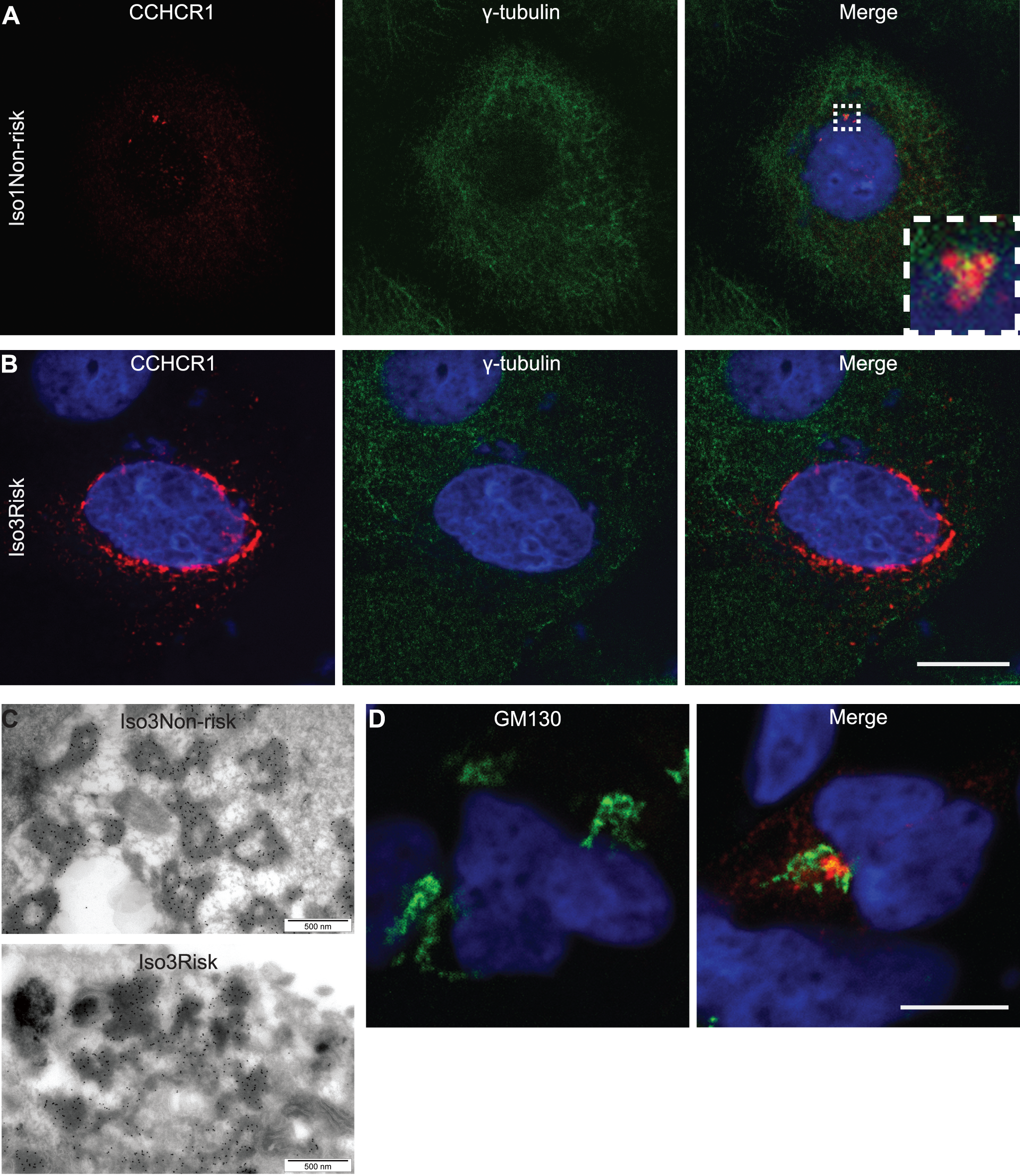

Supplement: Figure S2 — Expression pattern of CCHCR1 in transfected cells. (A) CCHCR1 (red) colocalizes with γ-tubulin (green) also in transiently transfected primary keratinocytes. (B) The Iso3Risk shows stronger perinuclear staining than the other constructs. Immunofluorescence staining of transfected cells with CCHCR1 antibody suggests that the cytoplasmic granules are smaller when formed by the Iso3Non-risk or the Iso1 constructs than by the Iso3Risk construct. (C) IEM of transfected COS-7 supports the observation; the gold label in the cytoplasm is observed as clusters that are larger in the Iso3Risk than Iso3Non-risk cells. (D) Cis-golgi marker GM130 (green) surrounds and has partial colocalization with the centrosomal CCHCR1 (red) in stably transfected CCHCR1 cell lines (here shown Iso1Non-risk). DAPI stained nuclei are shown in blue. Scale bar 10 µm. (TIF) [file pone.0049920.s002.tif]

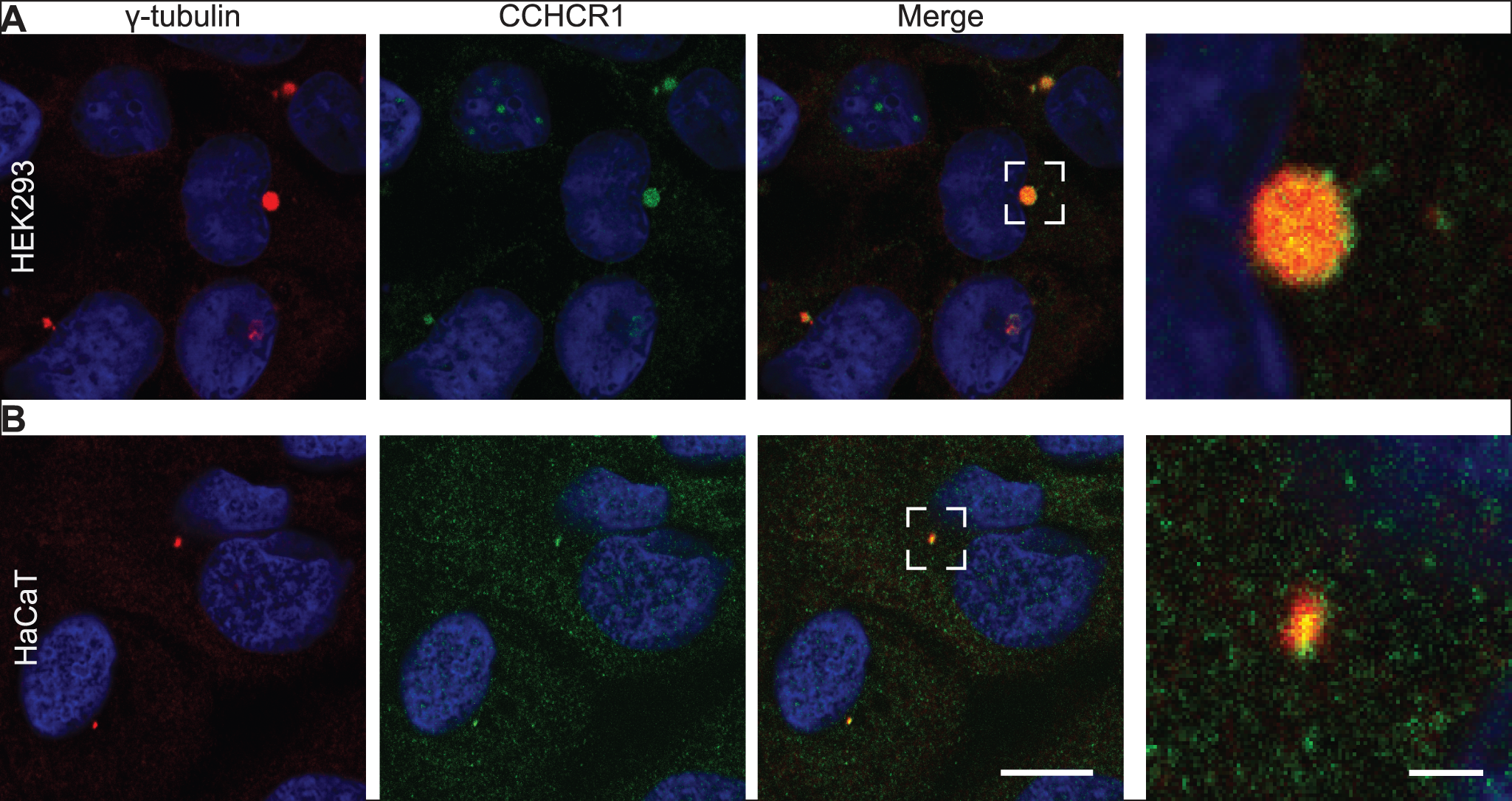

Supplement: Figure S3 — Localization of endogenous CCHCR1. Immunofluorescence staining shows that the endogenous CCHCR1 localizes at the centrosome in (A) HEK293 and (B) HaCaT cells. Cells were stained with CCHCR1 (green) and γ-tubulin (red) antibodies. The last picture shows magnification of the centrosomal region, marked in the merge channel pictures. DAPI stained nuclei are shown in blue. The scale bars are 10 µm and 2 µm (the magnified picture). (TIF) [file pone.0049920.s003.tif]

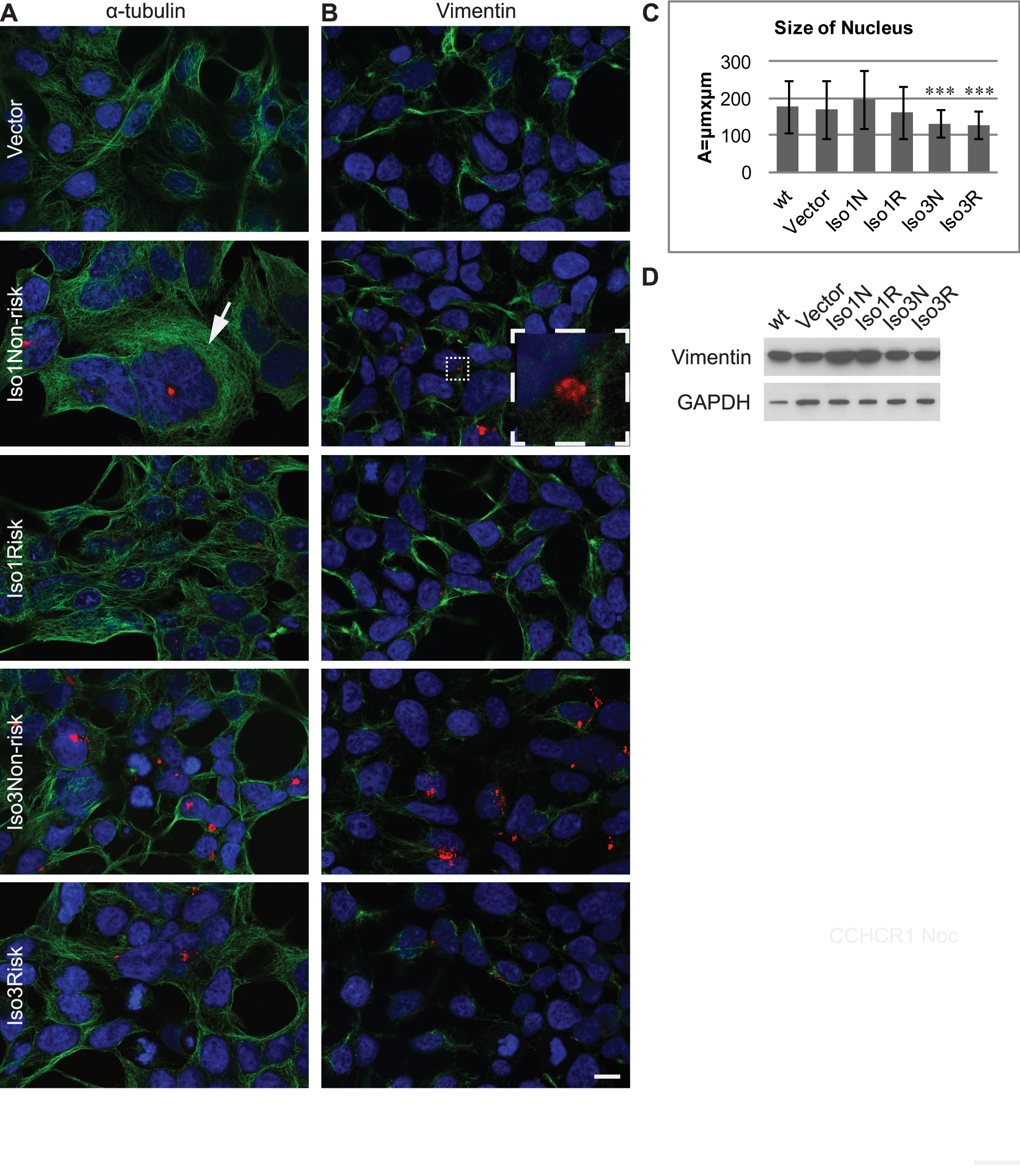

Supplement: Figure S4 — Morphological and cytoskeletal differences in stably transfected CCHCR1 cell lines. Comparison of all four CCHCR1 and control cell lines stained with antibodies against (A) α-tubulin and (B) vimentin. The stainings (green) reveal alterations in the cell morphology and cytoskeleton of cells expressing different CCHCR1 isoforms (red). (A) The α-tubulin staining pattern is otherwise unchanged in CCHCR1 overexpressing cells. Especially the Iso1Non-risk cell line exhibits multinuclei (indicated with an arrow). (C) The average size of the nuclei is significantly smaller in the isoform 3-expressing cells when compared to the vector-control cell line and even more significant when compared to Iso1Non-risk cells. Asterisks show significant P-values calculated with values from the vector-control cell line and the representative cell line (*<0.05>**<0.01>***<0.001). (B) Vimentin expression and organization is disturbed in both Iso3Non-risk and -risk cell lines as shown by immunofluorescence. (D) Immunoblotting with vimentin antibody, however, lacks evidence for major changes in expression between stable CCHCR1 cell lines. A magnification of the centrosomal region from Iso1Non-risk cells with the vimentin staining (B) shows that the CCHCR1 is not surrounded by a vimentin cage, which is typical for aggresomes. DAPI stained nuclei are shown in blue. Scale bar 10 µm. (TIF) [file pone.0049920.s004.tif]

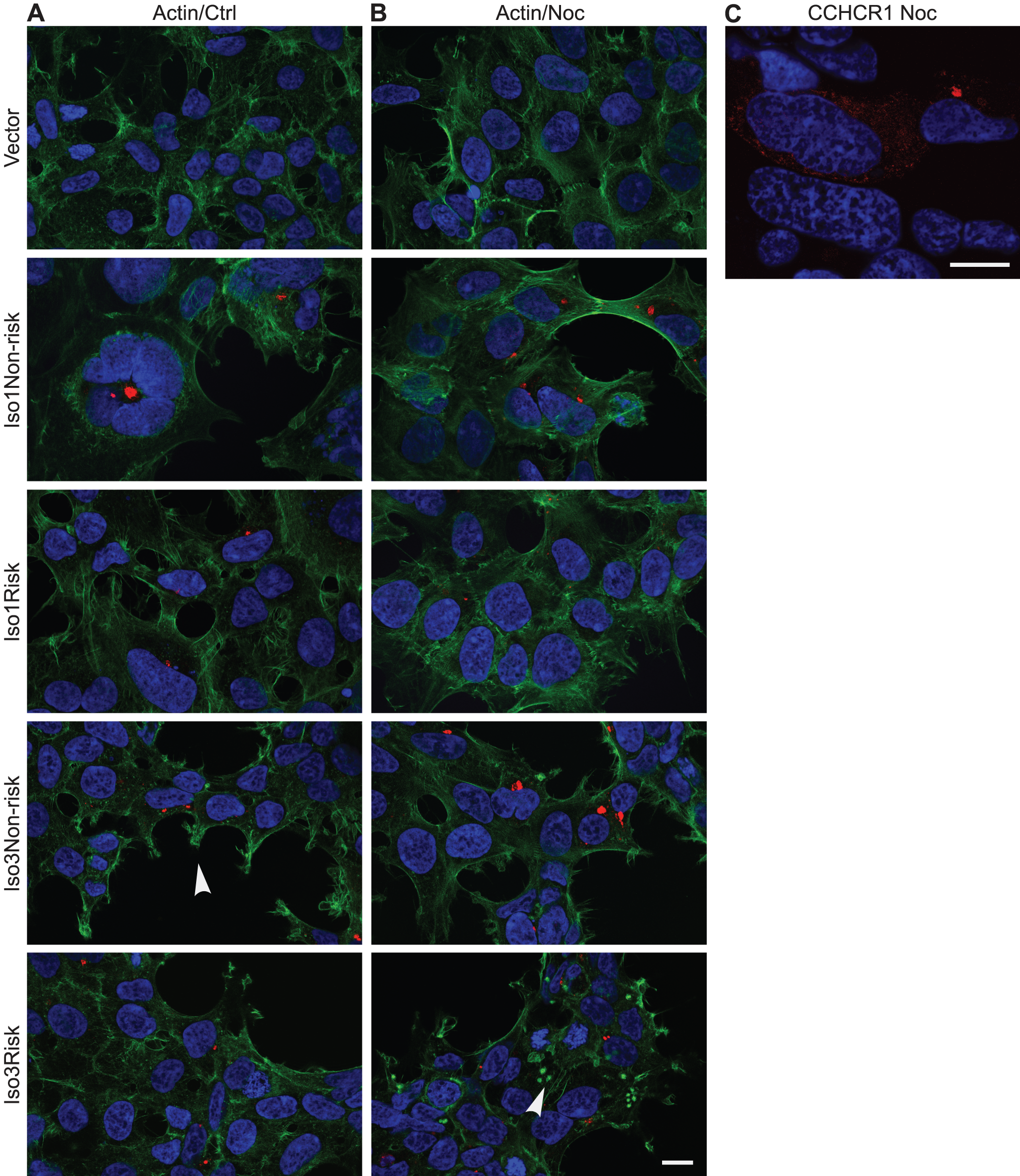

Supplement: Figure S5 — Microtubule disturption shows altered organization of the actin cytoskeleton in CCHCR1 Iso3Risk overexpressing cells. (A) Phalloidine staining shows that isoform 3-expressing cells exhibit more pseudopodia with actin rich tips, indicated by an arrowhead (Iso3Non-risk, Ctrl). (B) The most obvious change in actin organization is observed in the Iso3Risk cells, after treatment with nocodazole (Noc); actin forms punctate staining resembling podosome-like structures, indicated by an arrowhead (Iso3Risk, Noc). (C) The localization of CCHCR1 is partially changed after the disruption of microtubule network with nozodazole. DAPI stained nuclei are shown in blue. Scale bar 10 µm. (TIF) [file pone.0049920.s005.tif]

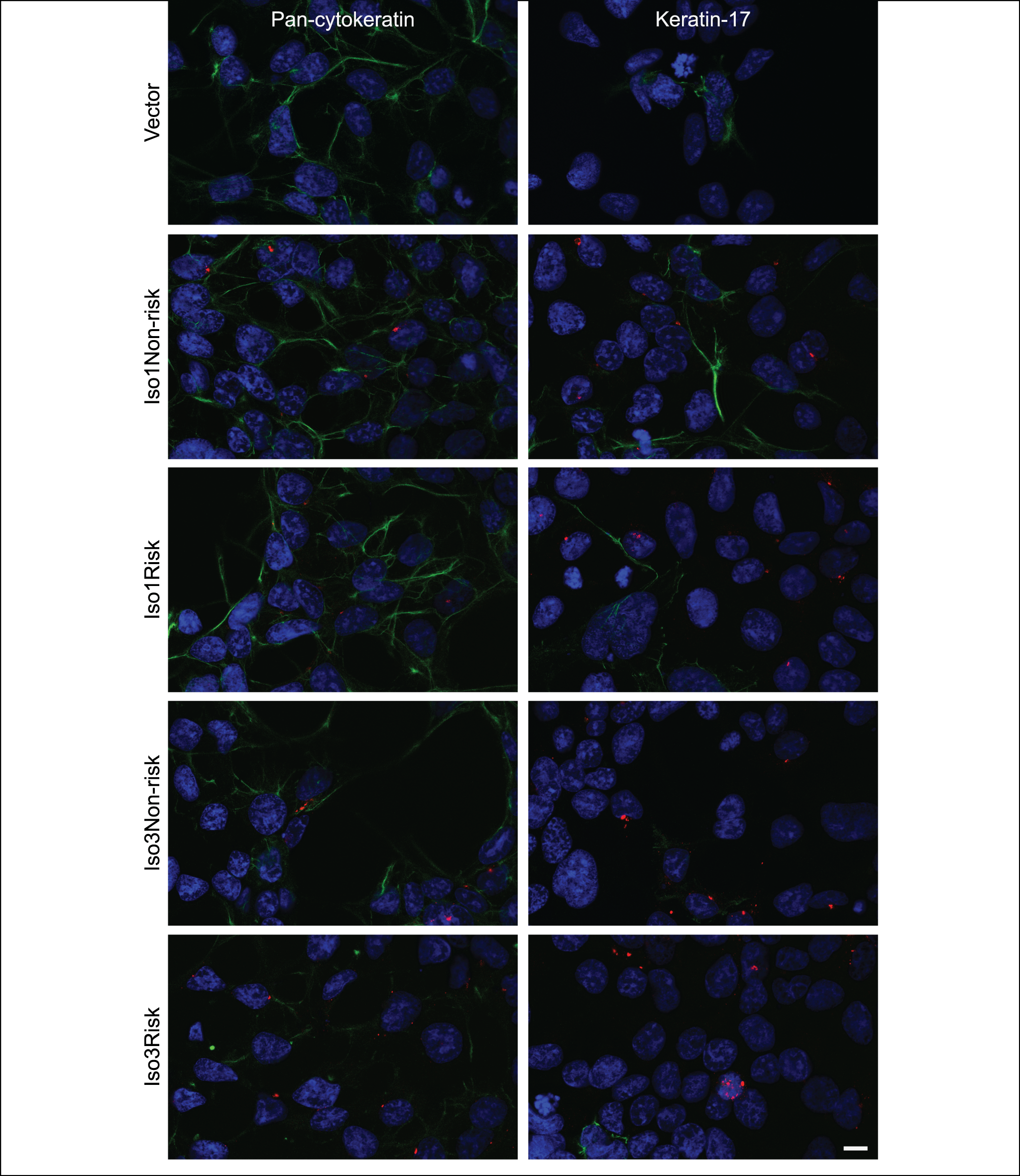

Supplement: Figure S6 — CCHCR1 affects the expression of cytokeratins. Immunofluorescence staining of (A) pan-cytokeratin and (B) keratin 17 in all four CCHCR1 overexpressing cell lines and vector control cells. (A) The overall expression of cytokeratins is reduced especially in the Iso3Risk-expressing cells. (B) Iso1Non-risk-expressing cells show an increase in the expression of keratin 17. DAPI stained nuclei are shown in blue. Scale bar 10 µm. (TIF) [file pone.0049920.s006.tif]

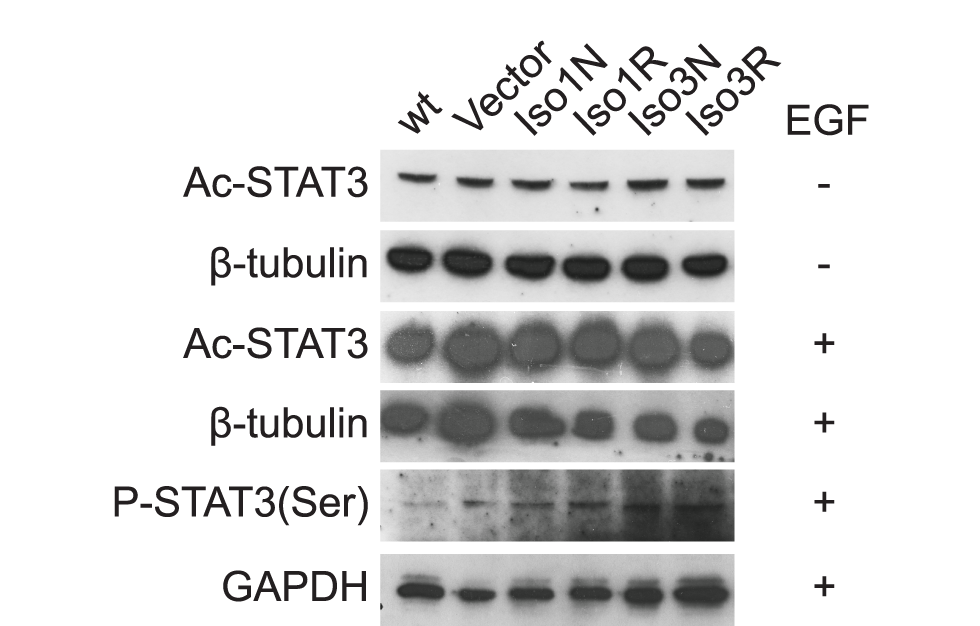

Supplement: Figure S7 — Overexpression of CCHCR1 in stable cell lines does not affect STAT3 serine 727 phosphorylation or lysine 685 acetylation. Staining with an antibody for β-tubulin or GAPDH was used to control sample loading on SDS-PAGEs. (TIF) [file pone.0049920.s007.tif]
